# Sleep increases firing rate modulation during interictal epileptiform discharges in mesial temporal structures

**DOI:** 10.1093/braincomms/fcag130

**Published:** 2026-04-30

**Authors:** Stephen Whitmarsh, Vi-Huong Nguyen-Michel, Katia Lehongre, Bertrand Mathon, Claude Adam, Virginie Lambrecq, Valerio Frazzini, Vincent Navarro

**Affiliations:** Sorbonne Université, Institut du Cerveau - Paris Brain Institute - ICM, Inserm, CNRS, APHP, Pitié-Salpêtrière Hospital, Paris 75013, France; Epilepsy Unit and Reference Center for Rare Epilepsies, ERN EpiCARE, AP-HP, Pitié-Salpêtrière Hospital, Paris 75013, France; Sorbonne Université, Institut du Cerveau - Paris Brain Institute - ICM, Inserm, CNRS, APHP, Pitié-Salpêtrière Hospital, Paris 75013, France; Sorbonne Université, Institut du Cerveau - Paris Brain Institute - ICM, Inserm, CNRS, APHP, Pitié-Salpêtrière Hospital, Paris 75013, France; Department of Neurosurgery, AP-HP, Pitié-Salpêtrière Hospital and Sorbonne Université, Paris 75013, France; Epilepsy Unit and Reference Center for Rare Epilepsies, ERN EpiCARE, AP-HP, Pitié-Salpêtrière Hospital, Paris 75013, France; Sorbonne Université, Institut du Cerveau - Paris Brain Institute - ICM, Inserm, CNRS, APHP, Pitié-Salpêtrière Hospital, Paris 75013, France; Epilepsy Unit and Reference Center for Rare Epilepsies, ERN EpiCARE, AP-HP, Pitié-Salpêtrière Hospital, Paris 75013, France; Sorbonne Université, Institut du Cerveau - Paris Brain Institute - ICM, Inserm, CNRS, APHP, Pitié-Salpêtrière Hospital, Paris 75013, France; Epilepsy Unit and Reference Center for Rare Epilepsies, ERN EpiCARE, AP-HP, Pitié-Salpêtrière Hospital, Paris 75013, France; Sorbonne Université, Institut du Cerveau - Paris Brain Institute - ICM, Inserm, CNRS, APHP, Pitié-Salpêtrière Hospital, Paris 75013, France; Epilepsy Unit and Reference Center for Rare Epilepsies, ERN EpiCARE, AP-HP, Pitié-Salpêtrière Hospital, Paris 75013, France

**Keywords:** epilepsy, multi-unit, intracranial, sleep, interictal

## Abstract

Epileptic seizures and interictal epileptiform discharges are strongly influenced by sleep and circadian rhythms. However, human data on the effect of sleep on neuronal behaviour during interictal activity have been lacking. We analysed EEG from eight epileptic patients implanted with macro and micro electrodes in mesial temporal structures. Sleep staging was performed on polysomnography and video-EEG. Automated detection identified thousands of interictal epileptiform discharges per patient. Both their rate and amplitude increased with deeper stages of non-rapid eye movement sleep. Single- and multi-unit firing rates were often temporally coupled with local field potentials, exhibiting increased firing during the spike and decreased activity during the following slow wave. These time-locked firing rate modulations were shown to increase during deeper stages of non-rapid eye movement sleep. Furthermore, neuronal background activity showed a decrease in firing rate, bursting and regularity with deeper stages of non-rapid eye movement sleep.

## Teaser

Whitmarsh et al. report that deeper non-rapid eye movement sleep increases interictal epileptiform discharge rates, amplitudes, and neuronal firing rate modulation. Using long-term intracranial recordings, they show that background firing rates, bursting, and firing regularity decrease with sleep depth, revealing sleep’s critical role in shaping interictal dynamics in epilepsy patients.

## Graphical Abstract

**Graphical Abstract:**
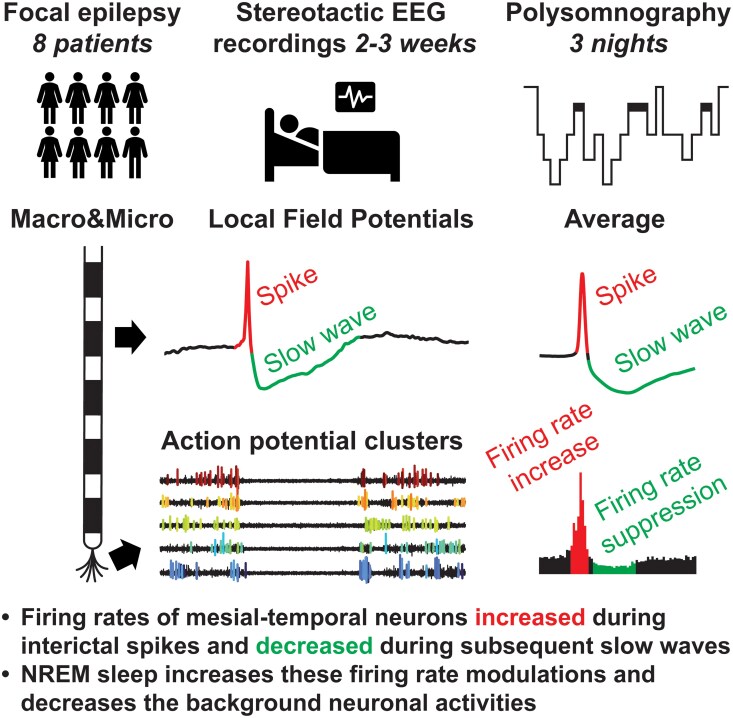

## Introduction

Early modern research recognized that epileptic activity occurs in circadian and ultradian cycles.^1,2,3^ Since the advent of electroencephalography (EEG) and polysomnography (PSG), it is now known that many types of epilepsy (focal and genetic generalized) have the highest seizure preponderance during light non-rapid eye movement (NREM) sleep, and the lowest during rapid eye movement (REM) sleep.^4,5,6,7,8,9,10,11,12,13^ In temporal epilepsy, focal seizures were also found to be more likely to evolve into bilateral tonic-clonic seizures during sleep than during wakefulness.^6,8,9^ In addition, sleep deprivation is the most common trigger for “breakthrough seizures”^14,15,16,17,18^ and seizures in the epilepsy clinic,^19,20^ regardless of the type of epilepsy or the epilepsy syndrome. Furthermore, sleep disorders such as sleep apnoeas, that strongly disturb the quality of sleep, likely worsen epilepsy.^21,22^

Interictal epileptiform discharges (IEDs) are brief non- or pauci-symptomatic events observed in the EEG of patients predisposed to spontaneous seizures.^23,24,25^ IEDs are known to be strongly influenced by sleep and circadian rhythms. The first demonstration that the rate of IED occurrence increases during sleep, even when the waking EEG appears normal, was provided by early work in the field.^26^ An increase in IEDs during drowsiness or sleep was later reported in certain patients using invasive EEG recordings.^27^ Since then, IEDs have been consistently found to occur more often during sleep.^28,29,30,31,32,33,34,35,36,37^ Indeed, long-term recordings have found robust 24 h periods, peaking between midnight and 5pm.^35,38,39^ Generally, deeper stages of non-REM (NREM) sleep seem to promote interictal activity, while lighter stages may promote seizures,^9,11^ especially in temporal lobe epilepsy.^28,33,34^ The average IED rate is lower in rapid eye movement (REM) sleep than in wakefulness, and higher in slow wave sleep (SWS) than in REM.^40^ Furthermore, IED rates are positively correlated with power in the delta (1 Hz to 4 Hz) frequency range.^29,41,42^

IEDs are understood to be generated by synchronous neuronal discharges, occurring during depolarization of the neuronal membrane,^43,44,45^ consistent with paroxysmal depolarizing shifts (PDSs) studied in animal models of epilepsy, in which a large depolarization phase is followed by a long hyperpolarization.^46,47^ In humans, micro electrode recordings have shown that firing rates increase during the interictal spike, and are subsequently reduced (and often completely silenced) during the subsequent slow wave.^45,48,49,50^ The effect of sleep on seizures and IEDs are hypothesized to be due to enhanced brain synchronization during SWS, and decreased synchronization during REM.^51,52^ Consistent with sleep-related changes in cortical excitability, human cortical and hippocampal neurons have been shown to have a higher propensity for bursting during NREM, with the lowest bursting rates during REM.^53,54,55^ Although these findings provide a pathophysiological model on the level of single unit and population activity, no studies have yet demonstrated an effect of sleep on neuronal firing behaviour during IEDs in human data.

Several studies have shown the clinical potential of basic morphological metrics of IEDs. Aanestad and colleagues reported a statistically significant reduction in the amplitude of spikes and slow waves with increasing age in patients with temporal lobe epilepsy.^56,57^ Increased IED amplitudes during sleep and after awakening have also been anecdotally reported in auto-limited epilepsy with centrotemporal spikes (BECTS).^58,59,60,61^ Subsequently,^62^ observed that the highest spike amplitude and shortest spike duration were often localized within 2 cm of the seizure onset zone, in a cohort predominantly composed of temporal lobe epilepsy patients. Spikes during REM sleep have been reported to often have lower voltage than those observed during wakefulness or NREM sleep,^34,41^ and that their sharpness and amplitude fluctuate during NREM.^41^ However, neither study provided quantification or statistical comparisons between sleep stages. A statistically significant increase in the spike amplitude during NREM compared to REM or wakefulness has been reported elsewhere, but based on a very limited number of observations.^63,64^

Recent investigations into spike morphology using a feature extraction approach revealed amplitude differences between brain regions and the seizure onset zone (SOZ).^37,65^ A non-significant trend towards increased amplitudes during deeper stages of sleep was observed, but only within the SOZ in medial brain regions. Taken together, although previous studies suggest that IED amplitudes may increase with sleep depth, consistent and statistically robust comparisons of IED amplitudes across all sleep stages remain lacking.

The current study investigated the influence of sleep on short and long-term dynamics of interictal activities, i.e. on time-locked neuronal activity during interictal events as well as on baseline activity without interictal events. For this purpose we analysed recordings from intracerebral electrodes containing both macro and micro contacts implanted in the hippocampal-amygdala complex of patients with focal epilepsy. An automatic template matching procedure was used to objectively identify IEDs in continuous long-term (2–3 weeks) recordings. Implanted micro electrodes allowed for the recording of neuronal action potentials during the full sleep and wake cycle. Polysomnography allowed detailed multilevel analyses during different stages of sleep.

We expected deeper stages of NREM sleep to increase the rate of IEDs, as well as their amplitude, while increasing background firing rates and bursting propensity. During the IEDs, we expected to find increased neuronal firing rates during IED spikes, and decreased neuronal firing rates during subsequent slow waves. Importantly, we expected these firing rate modulations during IEDs to increase with deeper stages of NREM sleep compared to REM and wake. Finally, we explored the effect of sleep on the regularity of neuronal firing with CV2.^66^

## Methods and materials

### Patients

Eight drug-resistant focal epilepsy patients were selected from our database,^67^ with the following criteria: (i) Implantation of macroelectrodes targeting the mesial temporal structures, (ii) micro electrode recordings with visible MUA, and (iii) polysomnography recordings during the first three nights. Dosages of anti-seizure medication were maintained during all recordings, consistent with our clinical protocol. See Table 1 for clinical details.

**Table 1:** Clinical summary

Patient	Sex	Age	Onset	Type	MRI	PET	SPECT	ASM	Implantation	SOZ	Surgery
1	F	18	10	FSLA, FTBS	Normal	Mesial prefrontal cortex (R)	No localization	LTG, OXC	Fronto-Temporal (R)	Mesial prefrontal cortex (R)	Yes
2	M	25	14	FSLA	Normal	Temporo-polar and temporo-mesial (L)	Amygdala, anterior insula, and frontal opercular cortex (L)	ESL, TPM, PER	Temporo-Fronto-Insular (L)	Amygdalo-hippocampal region (L)	Amygdalo-hippocampectomy (L)
3	F	34	25	FSWLA, FSLA, FTBS	Right SH (already resected at implantation)	Not available	Not available	VPA, CBZ, LTG	Bi-temporal	Bitemporal. Previously SH. Independent SOC in hippocampus (L)	No
4	F	47	24	FSWLA, FSLA, FTBS	Normal	Temporal pole (L)	Temporo basal and Lateral T1 (L)	ESL, LTG	Bi-temporal	Temporal (L)	No
5	F	30	14	FSLA, FTBS	Temporo posterior PNH (R), temporal SNH and PMG	No localization	Temporo-polar cortex (R), PNH and PMG	LTG, ZNG, LCS	Temporo-Insular (R)	Multifocal	No
6	F	27	11	FSWLA, FSLA, FTBS	Occipital porencephalic region (L)	Anterior hippocampus (L)	Temporal (L) and occipital	LCS, LTG	Temporo-Occipital (L)	Unknown	No
7	F	21	14	FSWLA, FSLA	Parietal gliotic lesion (L)	Parietal, temporal pole and temporo-lateral (L)	Temporal pole (L)	LCS, LTG	Parieto-Temporal (L)	Parietal (L)	No
8	F	38	35	FSLA	Normal	Temporomesial (R)	Lateral temporal cortex (L)	LTC, VPA, CBZ	Temporal (R)	Amigdalo-hippocampal (R)	Amygdalo-hippocampectomy (R)

PET findings indicate regions of hypometabolism. Seizure onset zones (SOZ) are based on stereotactic electroencephalography, but not yet validated post-surgery. ASM: Antiseizure medication, CBZ: Carbamazepine, ESL: Eslicarabazepine, FBTCS: Focal to Bilateral Tonic-Clonic Seizures, FIAS: Focal Impaired Awareness Seizure, FSWLA: Focal seizures without loss of awareness, IEDs: Interictal Epileptiform discharges, LCS: Lacosamide, LTG: Lamotrigine, MRI: Indications from Magnetic Resonance Imaging, OXC: Oxicarbazepine, PER: Perampanel, PMG: Polymicrogyria, PNH: Periventricular Nodular Heterotopia, SNH: Subcortical Nodular Heterotopia, SOZ: Seizure Onset Zone, TPM: Topiramate VPA: Valproic Acid, ZNG: Zonisamide.

### Ethics approval and consent to participate

All patients gave their written informed consent. This study was supported by INSERM (projects C11-16 and C19-55) and approved by the local ethics committee (CPP Paris VI).

### SEEG recording

Patients were implanted with intracerebral depth electrodes (Ad-Tech^®^, Oak Creek, Wisconsin, USA), consisting of 4 to 8 platinum macroelectrode contacts, 2.41 mm long (Ø=1.1 mm). Each patient was also implanted with one to three Behnke-Fried type macro-micro electrodes (Ø=1.3 mm) that included eight additional micro electrodes and a reference wire (Ø=40 µm), extending from the tip of the electrode shaft.^68^ To ensure both electrode types sample the same neuronal population, micro electrode wires were trimmed to 2 mm in length, placing their tip within 3 mm of the deepest macroelectrode contact. The LFP recorded by the deepest macroelectrode reflects activity from an estimated 1 cm^3^ of neural tissue, overlapping with the much smaller volume sampled by the micro electrode.^69^ In a previous study, we demonstrated that this approach captures overlapping neuronal populations, as evidenced by a strong correlation between LFPs recorded from both macro- and micro electrodes.^70^

Trajectories, anatomical targets, number, type and the number of contacts were all determined according to the clinical practice and the epilepsy features of the patients. Implantation was performed in the Department of Neurosurgery of the Pitié-Salpêtrière Hospital using a Leksell Model G stereotactic system (Elekta, Inc., Norcross, GA) or using a robotic assistant device (ROSA^®^ Brain, Medtech, France). For more details and evaluation of the implantation procedures, see.^67,71^

**Figure 1:**
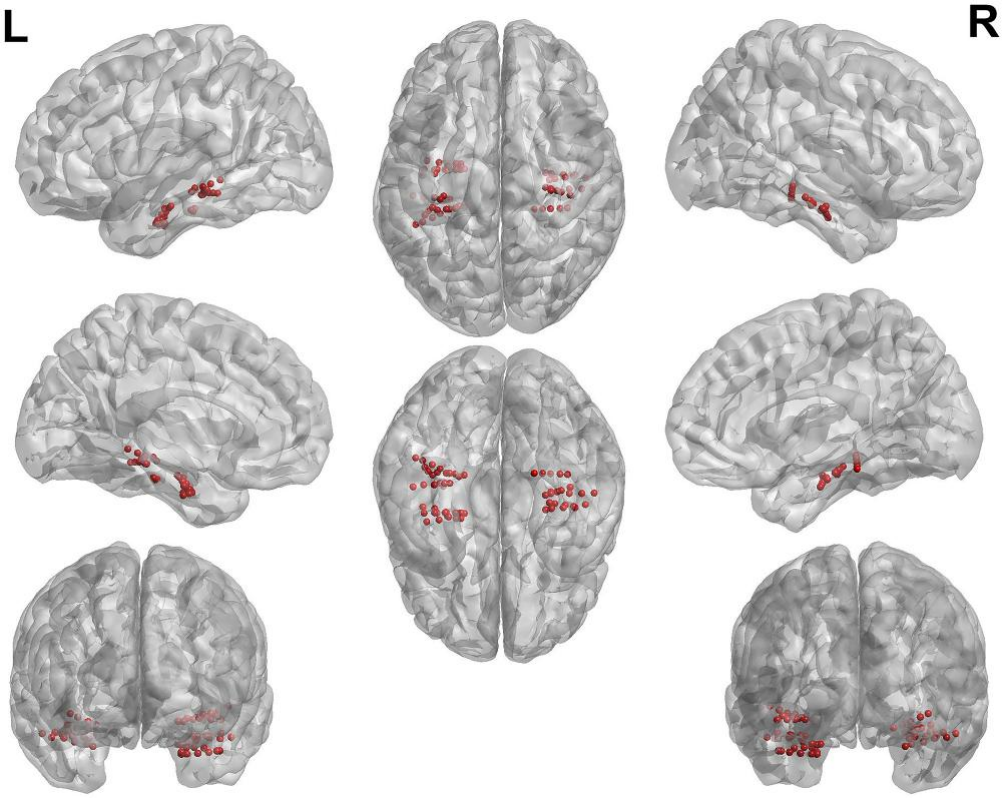
**Electrode localization.** Locations of each analysed electrode contact, transformed to Montreal Neurological Institute (MNI) space. See Supplementary Table 3 for MNI coordinates.

The locations of the anatomical electrodes after implantation were determined by VF (Supplementary Table 1), based on pre-implantation 3 T 3D-MRI, post-implantation 1.5 T 3D-MRI and post-implantation CT scan, combined using the EpiLoc plugin^72^ for 3D-Slicer,^73^ developed by the STIM engineering facility at the Paris Brain Institute. Spatial locations of the electrodes were automatically computed in native space using the EpiLoc plugin, and visualized (Fig. 1) using BrainNet Viewer,^74^ after transformation from to MNI-space (Supplementary Table 3) using the Freesurfer image analysis suite integrated in Epiloc.

### Polysomnography

During long-term video-EEG monitoring, video-EEG and polysomnography (PSG) were recorded simultaneously for the first three nights after implantation. When the first night was too disturbed, an extra night was scored and the first night was not analyzed. Following the American Academy of Sleep Medicine (AASM) 2017 criteria,^75^ PSG included at least three EEG channels: Fp2, C4 & O2, or Fp1, C3 & O1, referenced to linked mastoids M1 or M2, respectively, depending on the SEEG implantation site, as well as right and left electro-oculography (EOG) channels, chin electromyography (EMG) and electrocardiography (ECG). see Supplementary Fig. 4 & Supplementary Fig. 5 for examples. Sleep scoring was performed by VH-NM. A continuous period of two hours was labeled before sleep onset and after the final awakening, called *pre-sleep* (or *pre*) and *post-sleep* (or *post*), respectively.

Sleep quality varied among patients and nights (Supplementary Table 5). However, all patients had a sufficient amount of sleep per night (average = 8 h., range = 6.5–11.9 h.). Light sleep (S1) somewhat increased above normal (15% vs. <5), while deep sleep (average S3 = 20%) and REM (average = 19%) were normal.^76^ Hypnograms show a frequent sleep fragmentation (Supplementary Fig. 3), which might be explained by multiple factors, including the post-surgery and hospitalized condition.

### Analysis

Analyses were performed with a combination of FieldTrip functions,^77^ custom MATLAB scripts (Version 2020b, The Mathworks Inc., Natick, Massachusetts). All analysis scripts are available here.

### Artefact detection

In the first 3 days (72 h) of continuous recording, periods with movement artefacts or instrumentation noise in macro- or micro electrode recordings were manually annotated using software developed in-house (MUSE) for synchronous visualization of macro- and micro electrode signals.^67^ Data from one night (second) of a patient (patient 3) was replaced by a fourth night, due to excessive artefacts, and the polysomnography was then performed on that night as well.

### IED detection

**Figure 2:**
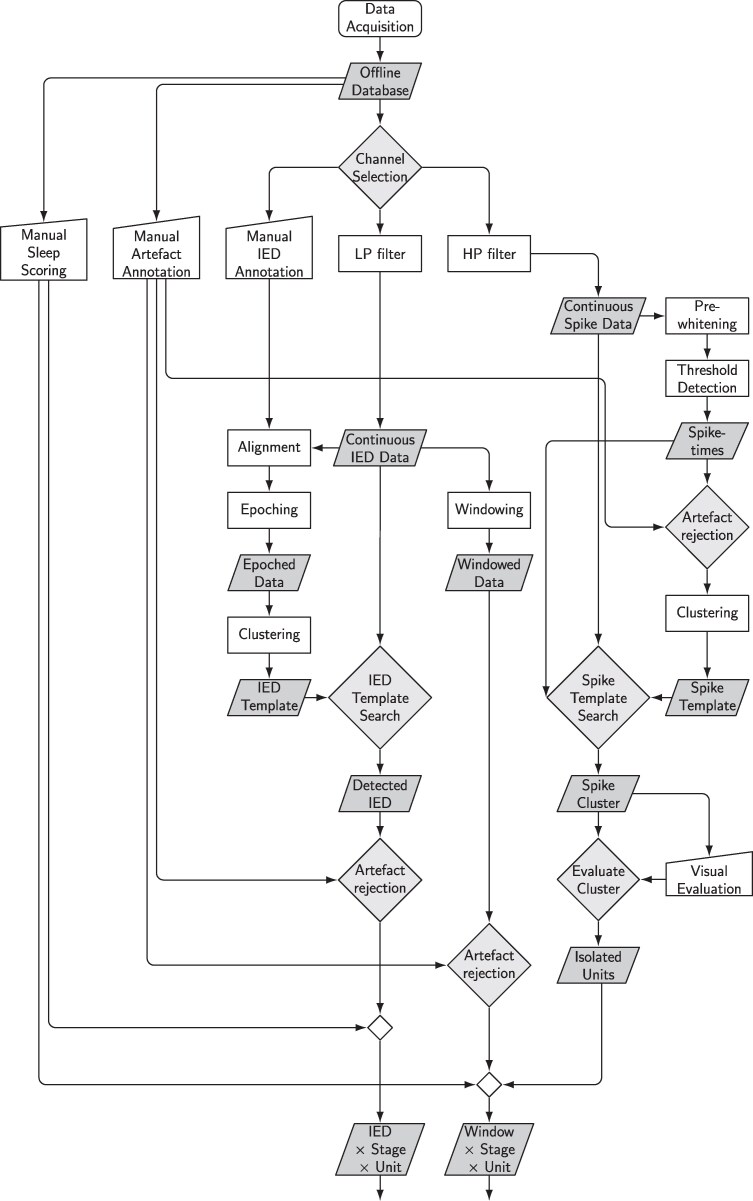
**Visual workflow.** Depiction of the LFP and spike analysis workflow. See the Methods section for details.

IEDs are fast (<200 ms) high-amplitude (>50 mV) EEG transients, defined as interictal spikes,^44^ habitually followed by a slow wave lasting several hundreds of milliseconds.^24,44^ For each patient, IEDs were manually annotated for the first 24 h, according to standard guidelines^78,79^ using in-house developed software (MUSE).^67^ IEDs were subsequently automatically detected for the full duration of the recording during (2–3 weeks) based on the following procedure (also see Fig. 2):

1. The time-courses (trials) were extracted (−500 ms to 1000 ms) at the original sample rate of 4 kHz according to manual annotations. Only the five deepest contacts were used, because these were A) located within the mesial temporal lobe structures showing the IEDs (Supplementary Table 1) and B) closest to the microelectrodes. IEDs were often reduced but still visible on the more superficial of these contacts but will still be informative for the detection approach.
2. Trials were temporally aligned (c.f.^70^) based on the cross-correlation between each trial and the average of all trials. This procedure was performed iteratively by calculating a new average after all trials were shifted to their peak cross-correlation lag, until either the cross-correlation did not improve or a maximum of 10 iterations was reached. To calculate the cross-correlation lag over channels simultaneously, signals from the five electrode contacts were concatenated over time.
3. Once aligned, the trials were clustered in six k-medoids clusters using the MATLAB *kmedoids* function (The Mathworks Inc., Natick, Massachusetts).
4. If a cluster contained less than 2.5% of the total number of trials in that patient, these trials were automatically removed from the clustering procedure and the k-mediods clustering was repeated.
5. Each cluster was aligned to the LFP average of the cluster, similar to (2), then re-averaged, resulting in a patient-specific Channel × Time IED template.
6. Normalized cross-correlation was calculated between each template and the original data using the MATLAB *normxcorr2* function (The Mathworks Inc., Natick, Massachusetts).
7. Patient-specific correlation thresholds were visually determined based on the distribution of correlation values over time. IEDs were then detected when the peaks in the correlation values exceeded the threshold, using the MATLAB *findpeaks* function (The Mathworks Inc., Natick, Massachusetts)
8. IED detections that overlapped within a ±50ms period were assigned to the template with the highest normalized cross-correlation value.

The above automatic IED detection procedure effectively dealt with the within-subject variability of IED morphologies by using six clusters rather than a single template, as well as their spatial distribution by using normalized correlation over five contacts. Only a single patient-specific parameter (z-threshold) was needed. Importantly, this parameter was unbiased with regard to sleep stages. The procedure was straightforward and allowed the visual inspection of templates. To test the performance of the automatic detection procedure, the hit and false alarm rates were determined using a tolerance of ±50ms to allow for small differences in timing between manual and automatic detection.

### Spike sorting

After selecting electrodes that visually showed multi-unit activity (MUA) activity, clusters of action-potentials were automatically detected and clustered using Spyking Circus,^80^ for three separate but contiguous 24 h recordings (see Supplementary Fig. 6 for an example of data recorded with microelectrodes during sleep). Data was first high-pass filtered as 300Hz using a 3rd order Butterworth filter, then temporally whitened. Action potentials were detected at a user-defined 6 to 9 median absolute deviation, depending on the signal-to-noise level of the recording. Action potentials that occurred during artefacted periods were ignored. A combination of density-based clustering and template matching algorithms was used to cluster the detected action potentials.^80^ Clusters that did not show a clear morphology of action potentials or that were not stable across the recording were discarded manually. Finally, the clusters were labeled as putative single-unit activity (SUA) when they reflected well-isolated activity, based on the inter-spike interval (ISI), the percentage of violations of the refractory period (RPV=ISI<1ms), and action potential morphology. Otherwise, the clusters were labeled multiunit activity (MUA).

### Time-locked analyses

For each IED, the average LFP amplitudes of the spike and slow wave were extracted at latencies based on the patient average, i.e. independently from any analysis of sleep stages. LFPs were first baseline-corrected at −0.15 s to −0.05 s. For the sharp (positive) peaks, the latency was defined as periods between −0.15 s to 0.15 s at which the average LFP exceeded half of the maximum amplitude. For slow (negative) waves, the latency was defined as periods between −0.15 s to 0.05 s at which the average LFP was less than half of the minimum amplitude.

Spike times were epoched and time-locked to time periods identical to the LFP. For each IED, peri-stimulus time histogram (PSTH) spike rates were calculated with a bin size of 10 ms. See Supplementary Fig. 7 for an example. To control for multiple comparisons and non-normal distributions of firing-rates, non-parametric cluster-based permutation tests^81^ were used to determine time-periods where firing-rates changed significantly from baseline (−0.3 s to 0.1 s). A threshold of p<0.01 (first-level t-test) was used to determine contiguous temporal clusters, after which a threshold of p<0.05 (one-sided correction) determined whether the clusters could be explained by permutation (sum of t values, n=10.000). Those clusters that significantly increased or decreased their firing rate time-locked to the IEDs were considered responsive. Clusters that showed spurious statistically significant results due to lack of data were manually changed to non-responsive. To increase the robustness of the PSTH spike rates during IEDs, templates were combined. Latencies for comparing the PSTH spike rates between sleep stages of the peak and slow wave were determined on the basis of the width at half-prominence of the average PSTH, i.e. independently from sleep stage. Pearson’s correlations between LFP and PSTH of each unit were calculated after downsampling LFP to the PSTH time axis ($\frac{1}{10\;\text{ms }}=100\;\text{Hz}$).

### Sliding window analyses

Analyses of continuous data were based on segmentation of the data into non-overlapping windows of 10 seconds for both LFP and spike data. The number of IEDs that occurred during these time periods was determined. If a window overlapped >50 with a sleep stage, it was labelled with the corresponding sleep stage. Windows that overlapped with an artefacted period were removed from further analyses. Oscillatory power was calculated for every window using a Hanning tapered fast Fourier transform (FFT). The 10 s windows provided a frequency resolution of $\frac{1}{T}=0.1\;\text{Hz}$. Slow wave activity (SWA)^82,83^ was defined as the mean power in the range of 0.1 Hz to 2.5 Hz, including the 0.5 Hz to 2 Hz range as suggested by the guidelines of the American Academy of Sleep.^84^ Delta activity was defined as the average power in the 2.5 Hz to 4 Hz range. For each window, the firing behaviour of the unit was explored. Besides firing rate analyses, inter-spike variability was determined by means of CV2.^85,86^ CV2 is a statistical metric used to assess the regularity of inter-event intervals, especially for analysing spike train variability. A CV2 value close to 0 indicates regular firing, 1 suggests random firing, while values greater than 1 indicate irregular or bursty firing patterns. Bursts were detected according to,^54,87^ using a cut-off inter-spike intervals <5 ms. Interspike intervals and firing rates were corrected for bursts by removing bursting action potentials beyond the first action potential in a burst.

### Statistics

Models of the relationships between physiological measures were tested using mixed-effects linear models,^88^ using the lmer R package.^89^ Tukey’s post hoc tests were performed using the emmeans R package.^90^ Mixed effects models were used to increase sensitivity while ensuring robustness of the findings. Fixed effects refer to fixed but unknown population parameters such as coefficients in the traditional linear model (LM) tested e.g. with a t test or ANOVA. Random effects often refer to effects at the individual or subject level that are included in the model to take into account the heterogeneity/variability of individual observations but are usually not of direct interest (Yu et al, 2021). In the current study these random effects include the variability over patients, nights, IED templates and units.

Statistical test were conducted with R,^91,92^ version 4.1.2. Circular uniformity was tested with the circular version of the Rayleigh Test of Uniformity from.^93^ Statistical plots were created with the ggplot R package.^94^ The R scripts for all statistical analyses and graphs can be found together with the analysis scripts here.

## Results

### IED detection

**Figure 3:**
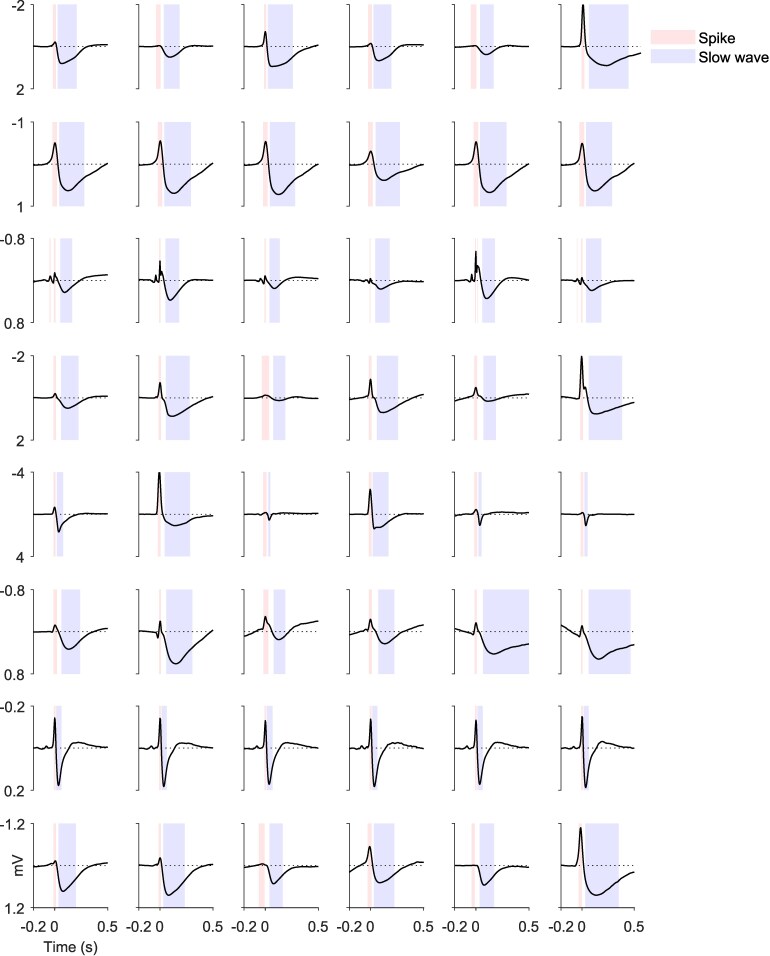
**Templates for automatic detection of interictal epileptiform discharges (IEDs) and statistical time intervals.** Each row represents the six templates of one patient (from top to bottom: Patient 1 to 8). Black lines show the template at the channel with the largest response over templates. Red overlays show the spike period [$<\frac{1}{2}\min(V)$], blue overlays the slow wave period [$>\frac{1}{2}\max(V)$]. Note the clinical direction of the current (top = negative).

Manual annotation of IEDs resulted in an average detection of 5667 IEDs per patient (2106 to 12 912) during the first 24 h (Supplementary Table 2). Automatic detection resulted in a similar average of 5618 IED detections per patient (3054 to 12 811) in the same 24 h, showing an average hit-rate of 98.0%, and an average false-alarm (false positive) rate of 6.5%. The total continuous recording time was on average 314 h per patient, i.e. just over 13 days, in which the automatic procedure detected an average of 50 271 IEDs per patient (9224 to 113 832, Supplementary Table 2). In all patients, the six templates showed morphologies consistent with spike-wave interictal discharges,^44,50,95,96^ with only modest variations in the amplitude of sharp peaks and slow waves (Fig. 3).

### Circadian distribution of interictal and ictal activity

**Figure 4:**
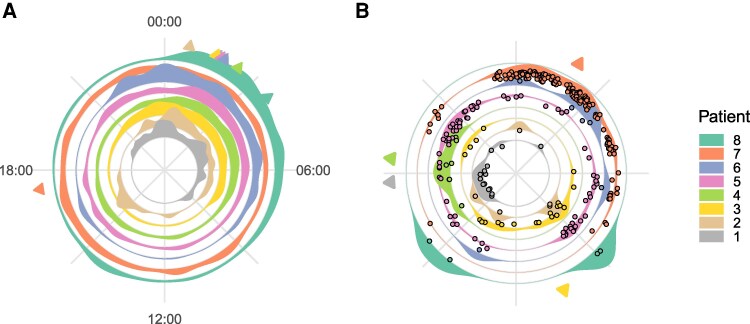
**Circadian interictal and ictal activities.** Polar representations of 24 h circadian distributions of (**A**) interictal activity, and (**B**) seizures. Of those patients that show a significant deviation from circular uniformity using a circular Rayleigh Test,^93^ have their mean angles of interictal density and seizure indicated with triangular markers. See Supplementary Table 4 for details.

IED rates showed a significant deviation from a uniform circadian distribution in all patients (Fig. 4A & Supplementary Table 4). Seven out of eight patients show a median phase between 01:00 and 03:30 at night, and the remaining patient presented a median phase at 17:25.

Seizure occurrence showed a significant deviation from a uniform circadian distribution in four of eight patients (Fig. 4B & Supplementary Table 4). In contrast to IED rates, the median phases of seizures were distributed throughout the day/night cycle. Of those patients who’s phase differed significantly from a flat distribution, the medium phase was found at 17:43, 10:31, 18:26 & 01:59.

### Increased IED rate with deeper stages of NREM sleep

**Figure 5:**
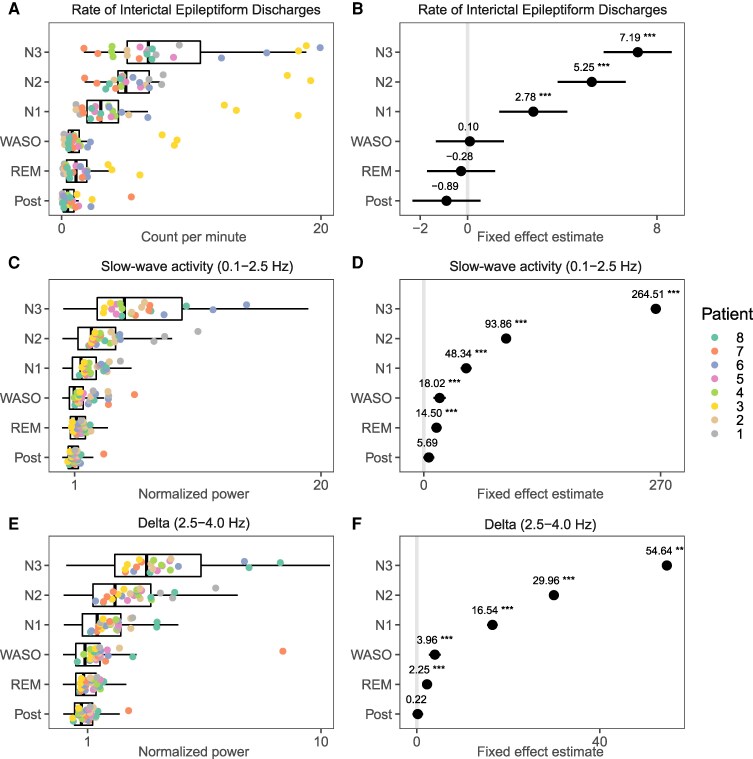
**Sleep modulation of spontaneous activities.** Summary statistics of interictal epileptiform discharge (IED) rates, slow wave activity (SWA) and delta power per sleep stage, with box plots overlaid by values representing averages per patient (coloured) and night. Tukey’s post hoc comparisons with the pre-sleep period were performed on mixed-effects linear models, with patient, template and night as random effects. See Supplementary Table 9 for details. **A** and **B** show the rate of IEDs relative to the pre-sleep period, and the fixed effects estimations, respectively. See Supplementary Table 6 for statistical tests. **C** and **D** show the power in the SWA band (0.1 Hz to 2.5 Hz) relative to the pre-sleep period, and the fixed effects estimations, respectively. See Supplementary Table 8 for statistical tests. **E** and **F** show the power in the delta band (2.5 Hz to 4 Hz) relative to the pre-sleep period, and the fixed effects estimations, respectively.

IED rates increased significantly from wake to sleep (Fig. 5A and B & Supplementary Table 6), showing higher IED rates with increasing depth of NREM sleep (S3>S2>S1>Wake). REM and wake did not differ significantly in IED rates.

### Increased IED rate with increased slow wave activity and delta power

For each patient, LFP data were epoched in 10-second non-overlapping intervals for the whole, and labelled according to the sleep stage, while excluding those that overlapped with IEDs or artefacts. Power in 0.1 Hz to 5 Hz was calculated in steps of 0.1 Hz, then averaged for each sleep stage, as well as over all sleep stages. Power at each frequency was then calculated relative to the pre-sleep period. Relative power showed a peak in the SWA frequency range (0.1 Hz to 2.5 Hz) which increased from 0.7 Hz in S3, to 0.8 Hz in S2, 0.9 Hz in S1, and 1.2 Hz in REM sleep (Supplementary Fig. 1). Mixed effects analyses verified that variance in both SWA and delta power were significantly associated with sleep stage (Fig. 5C-F, Supplementary Table 8 & Supplementary Table 9), with post-hoc comparisons confirming that both SWA and delta power increased with depth of sleep (Pre<REM<S1<S2<S3). Variations in IED rate were significantly associated with power in both the SWA (0.1 Hz to 2.5 Hz), and delta range (2.5 Hz to 4 Hz) (Supplementary Fig. 2 & Supplementary Table 7).

### Increased IED amplitudes with deeper stages of NREM sleep

**Figure 6:**
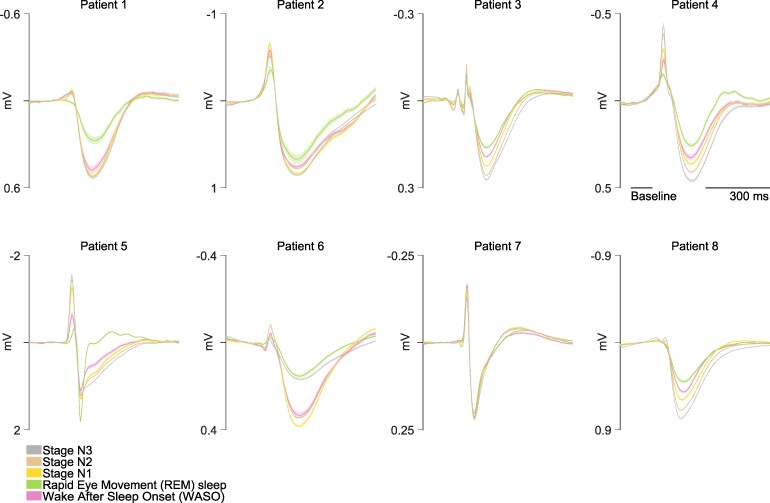
**Interictal local field potentials.** Average local field potentials (LFPs) of the interictal epileptiform discharges for each patient (panel) and sleep stage (colour). Standard error of the mean shown in lighter colour.

Figure 6 shows the LFP of all detected IEDs, averaged per sleep stage, showing that the magnitude of both the peak and the slow wave components increased with deeper stages of NREM sleep, in all but one patient (patient 7).

**Figure 7:**
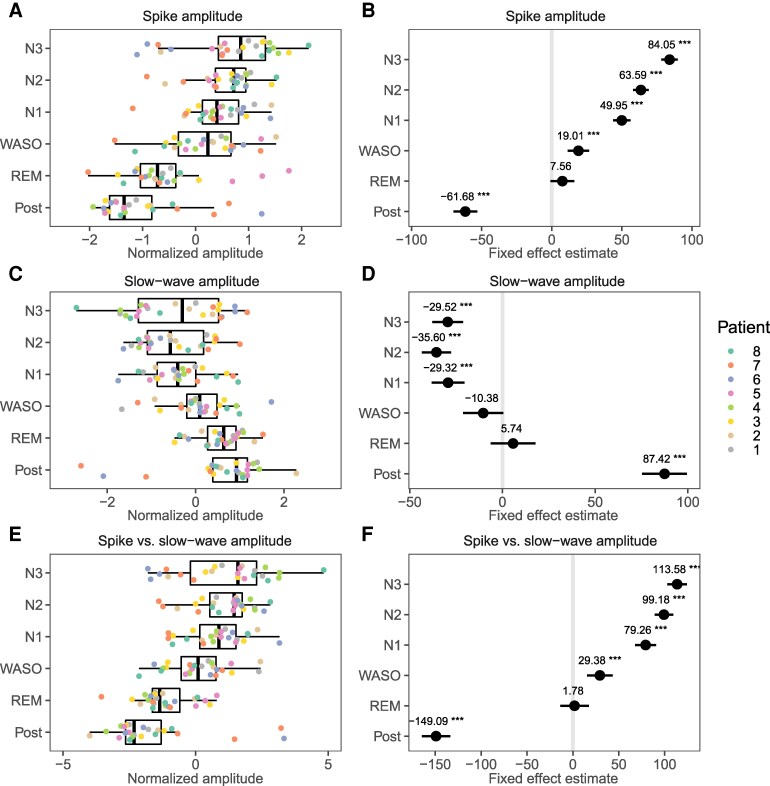
**Sleep modulation of spontaneous activity.** Summary statistics of interictal epileptiform discharge (IED) amplitude per sleep stage, with box plots overlaid by values representing averages per night, per patient (coloured). Tukey’s post hoc comparisons with the pre-sleep period were performed on mixed-effects linear models, with patient, template and night as random effects. See Supplementary Table 11 for details. **A** & **B** shows the average amplitude during the IED spike relative to those during the pre-sleep period, and the fixed effects estimations of the comparisons, respectively. **C** & **D** shows the average amplitude during the IED slow wave relative to those during the pre-sleep period, and the fixed effects estimations of the comparisons, respectively. **E** & **F** show the total deflection of the spike and wave ($V^{\;peak}-V^{SW}$) relative to the pre-sleep period, and the fixed effects estimations, respectively.

To statistically test the amplitude modulation of the spike and slow wave components of the IEDs, the average amplitude during their respective intervals, as determined earlier (Fig. 3), were entered into a mixed-effects analysis with patient and template as a random factor, and pre-sleep as the reference. Post-hoc comparisons allowed the evaluation of differences between sleep stages. The amplitudes of the IED spikes were increased in SWS compared to pre-sleep, WASO and REM, and increased progressively with increased depth within SWS (S3>S2>S1, Fig. 7A and B & Supplementary Table 11). Slow wave amplitudes decrease further (i.e. increased the deflection) during SWS compared to WASO and REM sleep, but were not significantly different within SWS stages. (Fig. 7C and D). When comparing the difference in peak and slow wave amplitudes ($V^{\;peak}-V^{SW}$), a clear gradual increase with decreasing arousal could be seen (Fig. 7E and F), with post-hoc tests showing a gradual increase of spike-wave deflection with increasing depth of sleep (S3>S2>S1). Neither the spike, wave or difference, was different between pre-sleep and REM.

### Firing rate modulation during IEDs increases during deeper stages of NREM sleep

**Figure 8:**
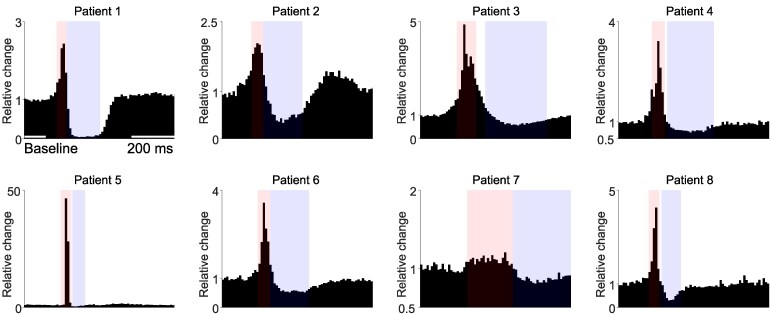
**Peri-stimulus time histograms (PSTH) of all responsive units, templates and sleep stages, separately for each patient.** For each patient, template and unit, firing rates were standardized by dividing the PSTH by the mean firing rate during baseline (indicated in the first panel). Red and blue overlays indicate intervals for statistical tests of the sharp wave and slow wave (Fig. 9 & Supplementary Table 13), based on the width at half-prominence of the patient-specific average PSTH plot shown here.

Over all patients and nights, 112 putative single units (SUA), and 131 multiunits (MUA) were extracted (Supplementary Table 10). In all patients, individual peri-stimulus time histograms (PSTHs) showed increases in firing rates time-locked to the IED spike, as well as more gradual and sustained decreases during the subsequent slow wave period (Supplementary Fig. 8–Supplementary Fig. 17). Some units only responded with an increase during the spike, while some responded with only a decrease during the slow wave. Many units showed both, however, and the average response showed a strong modulation consistent with the LFP (Fig. 8). Together, the majority of units were shown to be responsive to IEDs, i.e. showed significant changes in firing rates during IEDs versus baseline (SUA: 75.9%, MUA: 65.6%, Supplementary Table 10).

**Figure 9:**
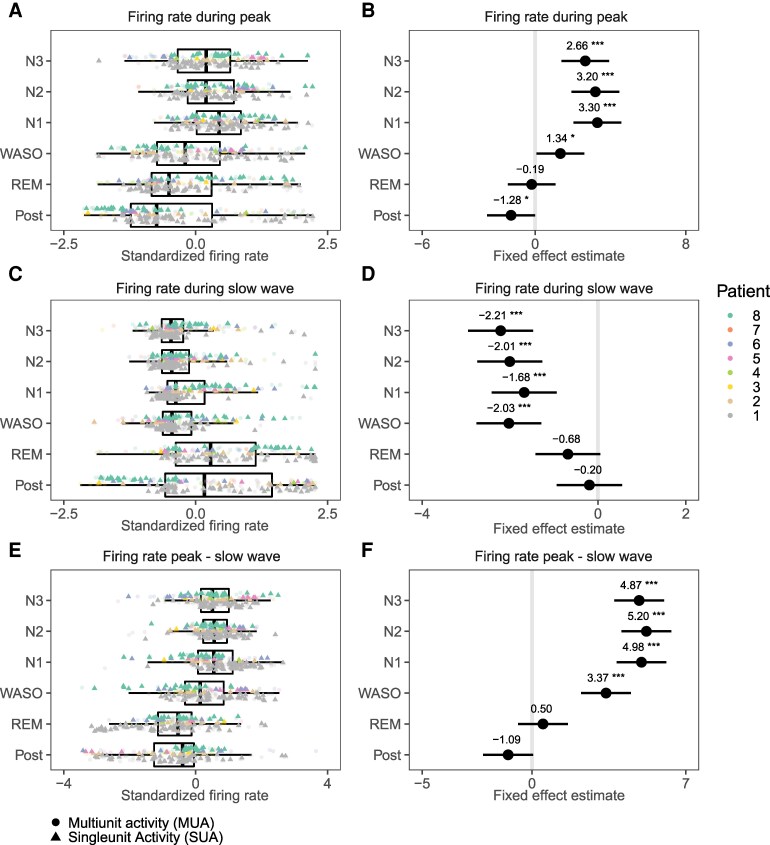
**Sleep modulation of interictal activities.** Unit firing rates during the spike and slow wave periods of the interictal discharges. Tukey’s post hoc comparisons with the pre-sleep period were performed on mixed-effects linear models, with patient, template and unit as random effects. See Supplementary Table 13 for details. **A**, **C** & **E** show box plots overlaid by values representing averages per patient (coloured) and unit. Pyramid shapes and dots represent single unit activity (SUA) and multi unit activity (MUA), respectively. For display purposes, values were normalized over all sleep stages. **B**, **D** & **F** show model estimates of fixed effects, including annotations for significant differences versus pre-sleep ($p<0.05^{.}$, $p<0.01^{*}$, $p<0.001^{**}$, $p<0.0001^{***}$).

To statistically test the effect of sleep stages on firing rate modulation during IEDs, the average firing rates of each unit during the spike or slow wave periods (Fig. 8) were entered into a mixed model. Firing rates during IED spikes increased significantly during NREM sleep (Fig. 9A and B, Supplementary Table 13) compared to the period before and after sleep. Firing rates during IED spikes were also slightly increased during wake periods after sleep onset (WASO). During the subsequent slow wave, NREM (as well as WASO) also significantly decreased firing rates compared to waking periods (Fig. 9C and D). REM was not found to affect firing rates during either the peak or slow wave. Finally, the normalized difference in firing rates between peak and slow waves (Fig. 9E and F) summarized the effect of NREM sleep on firing rate modulation during IEDs. During NREM sleep, as well as during WASO, the firing rate difference between peak and slow wave were increased. None of comparisons showed differences between S1, S2 and S3, and in all comparisons REM sleep did not differ significantly from waking periods.

### Unit background behavior changes with deeper stages of NREM sleep

Resting baseline activity of units, i.e. during periods in which no IEDs occurred, was investigated by epoching the first 72 h in 10-second non-overlapping intervals. These epochs were labelled according to the stage of sleep in which they occurred, and excluded if they overlapped with IEDs or artefacts.

**Figure 10:**
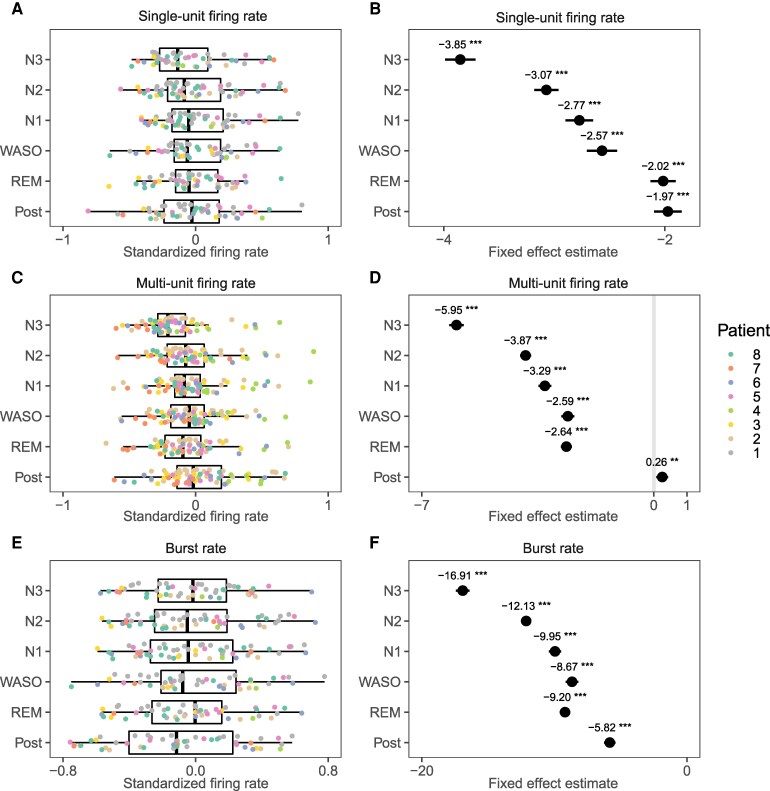
**Unit background activity (1/2).** Summary statistics of unit background activity during different sleep stages Tukey’s post hoc comparisons with the pre-sleep period were performed on mixed-effects linear models, with patient, template and unit as random effects. See Supplementary Table 12 for details. **A**, **C** & **E** show box plots overlaid by values representing averages per patient (coloured) and unit. For display purposes, values were standardized as relative change versus pre-sleep according to $\frac{x-Pre}{x+Pre}$). **B**, **D** & **F** show model estimates of fixed effects, including annotations for significant differences versus pre-sleep ($p<0.05^{.}$, $p<0.01^{*}$, $p<0.001^{**}$, $p<0.0001^{***}$). Only single unit activity (SUA) were included in the calculation of burst rates.

**Figure 11:**
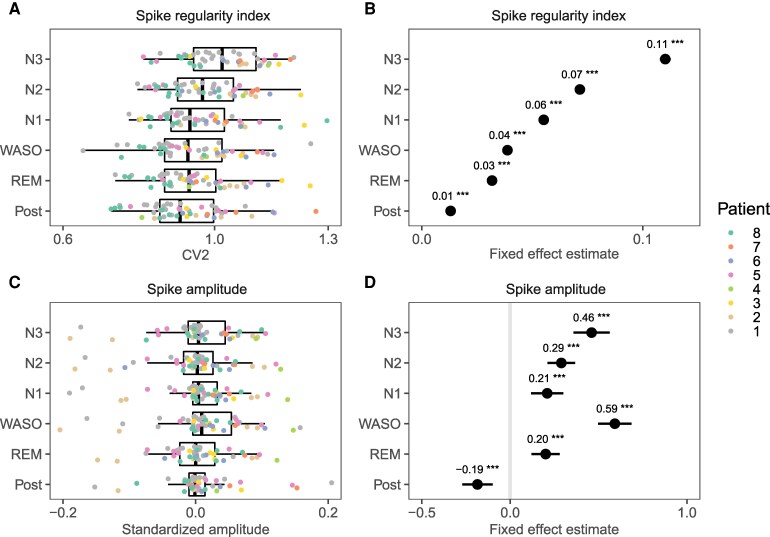
**Unit background activity (2/2).** Summary statistics of unit background activity during different sleep stages. Tukey’s post hoc comparisons with the pre-sleep period were performed on mixed-effects linear models, with patient, template and unit as random effects. See Supplementary Table 12 for details. **A** & **C** show box plots overlaid by values representing averages per patient (coloured) and unit. For display purposes, values were standardized as relative change versus pre-sleep: $\frac{x-Pre}{x+Pre}$). **B** & **D** show model estimates of fixed effects, including annotations for significant differences versus pre-sleep ($p<0.05^{.}$, $p<0.01^{*}$, $p<0.001^{**}$, $p<0.0001^{***}$). Only single unit activity (SUA) were included

Mixed models showed that SUA and MUA firing rates, as well as bursting rates decreased significantly with increasing depth of sleep, with post-hoc tests verifying a progressive decrease (pre>REM>S1>S2>S3, Fig. 10A-F & Supplementary Table 12). CV2 increased significantly, in an almost linear fashion until a large increase during S3 (S3>>>>S2>S1>Wake>REM), indicating increased regularity of action potential firing with deeper sleep stages (Fig. 11A and B). Finally, the amplitudes of the action potential increased significantly during all stages of sleep compared to both the pre and post period (Fig. 11C and D), in which post-hoc comparisons showed a progressive increase with S3>S2>S1.

## Discussion

### Interictal rate is under circadian influence

All eight patients showed a circadian distribution of IED rates, with seven peaking between 01:00 and 03:30 at night, consistent with previous reports (11:00–05:00 in,^38^ 02:21–04:12 in^35^ and one of the clusters at 04:00, 02:00, and 06:00 in^39^). While our population was heterogeneous with regard to the seizure onset zone (SOZ), including prefrontal, temporal, multifocal, and amydalo-hippocampal regions, all showed IEDs in mesial structures. Only one patient, with a SOZ in the left parietal region, did not show a nightly peak in IED rates from mesial structures. Although the sample size of our study prevent conclusions on the effect of the SOZ, previous studies have shown that the seizure onset zone (SOZ) and anatomical location do not effect circadian influences on interictal activity,^12,19,22,28,33,34,97,98,99^ but see.^36^

### Seizure occurrence is under circadian influence

Several patients showed a circadian influence on the occurrence of seizures. However, peaks of seizure occurrence were spread out along the day/night cycle. Patient 1, 3, 4 & 7 showed a circadian peak in the early evening, late morning, early evening, and night, respectively. This did not seem to be associated with a particular SOZ, which were frontal, temporal, temporal and parietal, respectively. Patient 5, and possibly Patient 3, showed a bimodal distribution, with a peak in the morning and evening, and a multifocal and temporal SOZ, respectively. The number of seizures of Patient 2, 6 & 8 were not sufficient to identify any potential circadian pattern. These results are in line with previous literature, showing circadian peaks in seizure occurrence^38,100,101,102,103,104,105^ with frontal lobe seizures generally peaking very early in the morning, and mesial temporal lobe seizures showing a more bimodal distribution, with a primary peak in the late afternoon and a secondary peak in the morning.^35,100,102,103,104,106,107,108^ The exact timing of the peaks are known to vary considerably between studies and patients, likely due to inconsistencies in protocols and analyses, as well as factors such as samples size, sample demographic, geographic location, seizure focus, and individual differences in circadian rhythms.^97,109,110^ Our results are consistent with varied circadian patterns in seizure occurrence, showing that temporal seizures display single and bimodal distributions throughout the day/night cycle, although the fact that our patient population had heterogeneous SOZ location might also have contributed to the variation in circadian distributions.

### IED rate and amplitude are modulated by sleep stage

Consistent with previous studies, the IED rate increased progressively during NREM sleep (S3>S2>S1>Wake)^28,29,30,32,33,34,35,36^ and no differences in IED rates between REM and wake,^34^ although the latter has been occasionally reported.^33,40^ Consistent with previous reports,^29,41,42,111^ we found that IED rates were positively correlated with power in the slow wave (0.1 Hz to 2.5 Hz) and delta (1 Hz to 4 Hz) frequency range.

The increase in IED rates during SWS and their suppression during REM sleep has been hypothesized to be due to increased brain synchronization during SWS and decreased synchronization during REM sleep.^51,52^ In fact, the same networks of cortical neurons involved in synchronizing thalamically generated delta oscillations during SWS may serve as the preferential substrate for epileptiform spike-wave activity.^112,113,114,115^

Of note, an inverse correlation between IED rate and delta power within the first NREM cycle was observed in 8 out of 10 patients with type 2 focal cortical dysplasia.^116^ While delta power was shown to be increased during the NREM cycle, IED rate was reported to decrease. This might be explained by the visual detection of IEDs which was based on a 3:1 peak-to-peak amplitude ratio to background activity. High-amplitude delta power during the NREM-cycle would therefore have decreased IED detection, resulting in an apparent negative correlation.

Several studies reported an increase in the spatial distribution of IEDs during SWS and a decrease during REM sleep.^10,13,34,51,52,64,117,118,119,120,121,122^ However, differences in IED amplitudes are rarely reported and in none of the aforementioned studies. An increase in IED amplitudes could result in increases in electrical field spread, which could be misinterpreted as an increase in spike propagation. Furthermore, the extent of the reported changes in the spatial distribution has generally been small and not unequivocally found. For example, in cases of mesial and neocortical temporal lobe epilepsy, interictal activity was found to shift from anterior to posterior and from mesial to lateral during sleep, while still remaining localized within the amygdalo-hippocampal complex^13^. Asano et al.^123^ found no effects of sleep on the spatial distribution, and^121^ found spatial distribution not to be predictive of seizure occurrence. Finally,^36^ found an increase in co-occurrence of different interictal sources during SWS, but only for neocortical spikes, not mesial-temporal spikes.

The current study analysed an very large number of IEDs (3500 to 12 811 per patient) from continuous data recorded over three day/night cycles and found clear evidence of an increase in IED amplitude during sleep. The LFP deflections of both the spike and slow wave were found to be gradually increased with deeper stages of sleep. In addition, both LFP deflections and neuronal firing rate were found to be lower in the post-sleep period compared to the pre-sleep period. The latter might be associated with the protective role of sleep through a mechanism of synaptic homeostasis.^55,124^ argue that prolonged sleep leads to a reduction in synaptic strength, along with decreased firing rates and neuronal synchrony. Such a sleep-related synaptic downscaling or “relaxation” may account for the changes observed in the post-sleep measurements. In conclusion, the current study found clear evidence that IED amplitude increases with deeper sleep stages and decreases post-sleep, potentially reflecting a sleep-related synaptic downscaling mechanism.

We found that firing rates during IED spikes were also slightly increased during wake periods after sleep onset (WASO). This finding may be related to the fact that WASO state is likely of lower alertness level than the diurnal waking state, as seen by^125^ who found 61% of WASO time occupied by slow eye movement, and indication of drowsiness and sleep onset.^126^ Our findings therefore indicate that neuronal firing patterns during WASO might resemble a transitional sleep-wake state rather than a (diurnal) wake state.

### Interictal activity modulates firing rates of the majority of neurons

Early studies found that some neocortical neurons discharged in time with interictal spikes.^127^ These have been termed “positive”^128^ or “involved” neurons,^129^ and were considered to reflect the intensity of epileptic activity. More recent studies have consistently found that a proportion of units increase their firing rate during the interictal spike, which is then reduced during the subsequent slow wave.^45,48,49,50,130^

Our results show a consistent increase and decrease of firing rate time-locked to the spike and slow wave of the IEDs in most patients. Units increased their firing rate sharply during, or right before, the upward flank of the sharp LFP spike, then reduced their firing rates to below baseline, highly consistent with the shape of the LFP slow wave. Only one patient (Patient 7) did not show fast and strong firing rate modulation during the IED spike. However, in the rest of the patients, the majority of units showed significant changes in firing rates versus baseline (SUA: 75.9%, MUA: 65.5%).

This high proportion of ”involved” units is somewhat higher than the 49% found by,^130^ the 48% reported by^49^ or the 40% found by.^48^ However, the experimental design and statistical inferences leading to these percentages differ greatly. The early work by^130^ was performed visually, and on fewer than 50 examples as indicated in their representative figures.^49^ excluded sleep periods and reported a large variability in the number of manually annotated IEDs per patient, ranging from 31 to 608. We analysed three 24-hour periods of continuous recordings using automatic IED detection, which resulted in between 2,106 and 12,912 IEDs per patient.

Furthermore,^49^ based statistical comparisons on average responses within five manually determined time periods relative to interictal peak, which would not allow for inter-patient differences in IED morphologies. Similarly,^48^ manually selected four time periods, in recordings ranging from 10 to 118 minutes, for a total of 863 IEDs. Unlike previous studies, the current study utilized a data-driven approach on a substantially larger dataset and accounted for interpatient variability in LFP morphology by identifying responsiveness through temporal cluster analysis. This analysis captured any time interval relative to IEDs where firing rates were consistent, allowing different time periods to be affected per patient. Clinical differences can also explain the higher rate of responsive neurons. In the studies by,^48,130^ clinical information was not provided.^49^ recorded from diverse regions (hippocampus, frontal cortex, lateral temporal cortex, and parieto-occipital regions), with 269 out of 363 units recorded from dysplastic cortex, 26 of cryptogenic origin, and the remaining 73 from various lesions. The present study included only patients with mesial temporal lobe epilepsy, without any MRI-detected lesions, and restricted to the hippocampal-amygdala complex, allowing better control of inter-subject variability. Furthermore, the hippocampal formation has anatomical and physiological mechanisms that promote neuronal synchronization, making this region highly prone to epileptic discharges. Finally, whereas^49^ used different recording techniques, including NeuroPort arrays, laminar micro electrode arrays, and AdTech electrodes, the current study only deployed the latter. Together, while our results may not generalize across all epilepsy types or brain regions, they show a remarkable consistency of firing rate behaviour in IEDs generated in medial temporal structures.

Together, our findings are consistent with the paroxysmal depolarizing shift (PDS) mechanism in which a large depolarization phase is followed by a long hyperpolarization found in animal,^46,47^ and consistent with *in vitro* studies on hippocampal slices from human patients with temporal lobe epilepsy.^49,131,132^

### Firing rates modulation increases with deeper stages of NREM sleep

While modulation of firing rates during IEDs has been established and replicated here, it is unknown whether this modulation is under the influence of sleep. Our results show for the first time that the firing rate during the sharp LFP peak is significantly increased during sleep compared to waking periods, while firing rates during the subsequent slow wave are significantly reduced.

The first part of the PDS depolarization phase is believed to be generated by intrinsic membrane conductances,^133^ and the later from feedback recurrent synaptic excitation mediated by AMPA and NMDA receptor subtypes, and glutamate receptor-coupled calcium conductances.^134^ The subsequent hyperpolarization represents GABA-mediated recurrent inhibition, as well as Ca2+-dependent outwards K+ currents. Our results suggest that sleep increases excitability and synchronization of neuronal populations, increasing both the probability of the occurrence of IEDs, as well the degree of de-facilitation by subsequent hyperpolarization.

### Neuronal baseline activity is modulated by sleep stages

Neuronal baseline activity, i.e. in the absence of IEDs, was investigated by means of firing rates, burst rates and CV2. These were calculated from a large number of non-overlapping 10-second time windows in 72 h, allowing robust estimations of conditional means within patients. By using mixed models, we dealt with both between-subject and within-subject variance. Firing rates, burst rates and CV2 were all shown to be lower under increasing depth of sleep, and especially pronounced during SWS. A reduction in the firing rate during SWS is consistent with the periodic suppression of neuronal firing due to SWA during NREM sleep, as shown in animal *in vivo* intracellular recordings.^53,55^ Although measures of regularity were not reported in these studies, decreased regularity with decreased depth of sleep could be explained by such “clumping together”^53^ of action potentials.

These studies, as well as our own findings, are in partial disagreement with,^54^ who reported a reduced firing rate in REM versus wake and SWS, a higher propensity for bursting during SWS versus wake and NREM, and increased variability (CV2) in SWS versus wake. However, these analyses were performed on only 23 neurons and, as the authors themselves noted, potential influences from interictal discharges were not considered. Another study of the same lab included a larger number of isolated neurons (72), and reported higher burst rates in MTL regions, but only during sleep (SWS and REM), and lacking consistent difference in firing rates between sleep stages or between epileptic versus non-epileptic regions.^135^

Although early human studies found indications of bursting in epileptic regions,^136,137^ others found no differences in firing patterns such as bursting between neurons in the epileptic focus or surrounding area.^127^ In fact, while some studies continued to associate bursting behaviour with epilepsy in both animal^138^ and human in vitro studies,^139,140^ micro electrode recordings in epileptic patients have so far returned contradictory results, showing both increased^135,141^ and decreased^87^ bursting in the SOZ of MTL patients.

### Automatic detection of IEDs

The current study exploited the possibility of analysing a very large number of IEDs by using automatic detection of IEDs. Many algorithms have been proposed for IED detection over the last decades,^142,143,144,145^ with recent implementations showing good, or better, performance than human experts.^146,147,148,149^ Recent examples include the use of adaptive morphological filters,^150^ signal envelope distribution modelling,^151,152^ convolutional neural networks (CNN) and deep learning,^153,154,155,156,157,158^ long short-term memory (LSTM) neural networks^159^ and generative adversarial networks (GANs).^160,161^ Two-step methods have been proposed to reduce the need to manually optimize parameters per different datasets.^65,162^ Furthermore, machine learning models need to be trained on large standardized datasets of annotated data to deal with the challenge of generalizing models over different patients. Such datasets currently do not exist, but are necessary to objectively compare the performance of the available machine learning approaches.^142,144,145^ Furthermore, the inherent unbalanced nature of interictal data, i.e. the small ratio of positive events within large periods of background activity, has only recently been addressed.^163^

The use of LFP templates provide a more robust and transparent alternative, and have been shown to outperform any classifier trained on morphological features.^164^ Template matching approaches have also been shown to perform very well even with high noise situations such as recordings taken from within an fMRI scanner.^165^ Some studies have used templates derived from databases,^166^ used patient-specific annotations,^167^ or performed clustering of patient-specific IEDs for rapid visual inspection and validation.^157^ In the current study, we chose to employ template matching based on patient-specific IED templates, extracted from visual annotations of 24 h. The approach showed to be highly effective, while remaining relatively straightforward. Importantly, the templates can be visually evaluated, in contrast to “black-box” approaches using machine learning. By expanding the IEDs into six templates per patient, potential variations in the morphology of IEDs were taken into account as well.

### Conclusion

The current study evaluated for the first time the influence of sleep stages on neuronal firing during interictal activity, by means of *in vivo* recordings from hippocampal structures in epileptic patients. This was achieved with a very large dataset of continuous recordings of several nights, and thousands of IEDs per patient. The IED rate as well as amplitude were found to be increased with deeper stages of NREM sleep. Neuronal firing rates were found to increase during the IED spike, and decreased during the slow wave. Importantly, these firing rate modulations during IEDs were increased with deeper stages of NREM (SWS) sleep, versus REM and wake. Finally, during background activity the neuronal firing rate, bursting rate and firing regularity were all shown to progressively decrease with deeper stages of NREM sleep. Together, this study builds on existing evidence that sleep enhances neuronal synchronization^168,169^ and that interictal epileptiform activity further entrains neuronal firing rates.

### Limitations

This study offers a comprehensive and robust approach to investigating how sleep influences interictal processes and modulates associated neuronal firing patterns. It employs methods designed to maximize information and statistical power from large datasets, despite the limited number of patients. However, this approach comes with certain limitations, both in terms of the scope of questions that could be addressed and the clarity with which the findings can be interpreted.

On the one hand, the limited number of patients and recorded brain regions limits the ability to address questions such as the influence of the seizure onset zone (SOZ), specific brain regions, epilepsy types, or the effects of medication as these require sufficient representation across different categories to be meaningfully addressed. For example, our recordings were done twice from the amygdala, five times from the entorhinal cortex, and seven times from the hippocampus, each in different patients (Supplementary Table 1). Therefore, the findings should be generalized with caution, as the study population does not fully reflect the diversity and complexity of the epileptic brain, focusing instead on the neural dynamics of interictal epileptiform discharges in the mesial temporal regions in focal epilepsy.

On the other hand, we were able to extract substantial amounts of data from each patient, enabling analyses that leveraged repeated measures within patients using mixed linear models for robust results. The strength of this approach lies in its ability to focus on comparisons of interest (fixed effects) while accounting for variability in factors not of interest (random effects), such as mean LFP amplitude or mean firing rates, which differ across patients, electrodes, and recording nights. However, this can sometimes complicate result interpretation, as the differences in averages across patients can deviate from the fixed effect estimates reported by the model, which account for these variabilities. This discrepancy is illustrated in Figs. 7, 8, 10 and 11 when comparing the left and right panels: large effect sizes shown in the model (right panels) cannot always be easily inferred from the displayed data (left panels).

The initial clustering before averaging of annotated IEDs was motivated by potential morphological variability of IEDs in individual patients. This potential diversity warranted a method capable of capturing waveform differences within patients in template-based detection. The variability within patients was found to be minimal, with clusters leading to nuanced templates that have a minimal impact on performance except for increasing robustness in reducing potential influences of artefacts and outliers. Downstream analyses were ultimately agnostic to the number of clusters used, as all detected IEDs were pooled following classification and modelled as random effects in the statistical analyses. A choice of k = 6 was empirically found to be sufficient, with no observed improvement in performance when varying k. This insensitivity suggests that the clustering step did not bias or constrain later stages of analysis. Therefore, future work could reasonably consider bypassing clustering altogether, particularly if the focus is on aggregated IED characteristics rather than subtype-specific features, with likely no effect on the findings.

## Supplementary Material

fcag130_Supplementary_Data

## Data Availability

All scripts are made available here. Anonymous electrophysiology data can be made available on reasonable request for research purposes, but cannot be publicly shared due to legal constraints.

## Author information

Stephen Whitmarsh, Data Analysis Core facility (RRID:SCR_026138), Sorbonne Université, Institut du Cerveau - Paris Brain Institute - ICM, Inserm, CNRS, APHP, Pitié-Salpêtrière Hospital, Paris, France

Correspondence: stephen.whitmarsh@icm-institute.org Vi-Huong Nguyen-Michel AP-HP, Pitié-Salpêtrière Hospital, Epilepsy Unit and Reference Center for Rare Epilepsies, F-75013, Paris, France Katia Lehongre Sorbonne Université, Institut du Cerveau - Paris Brain Institute - ICM, Inserm, CNRS, APHP, Pitié-Salpêtrière Hospital, Paris, France Bertrand Mathon Sorbonne Université, Institut du Cerveau - Paris Brain Institute - ICM, Inserm, CNRS, APHP, Pitié-Salpêtrière Hospital, Paris, France

AP-HP, Department of Neurosurgery, Pitié-Salpêtrière Hospital and Sorbonne Université, Paris, France Claude Adam AP-HP, Pitié-Salpêtrière Hospital, Epilepsy Unit and Reference Center for Rare Epilepsies, F-75013, Paris, France Virginie Lambrecq Sorbonne Université, Institut du Cerveau - Paris Brain Institute - ICM, Inserm, CNRS, APHP, Pitié-Salpêtrière Hospital, Paris, France

AP-HP, Pitié-Salpêtrière Hospital, Epilepsy Unit and Reference Center for Rare Epilepsies, F-75013, Paris, France Valerio Frazzini Sorbonne Université, Institut du Cerveau - Paris Brain Institute - ICM, Inserm, CNRS, APHP, Pitié-Salpêtrière Hospital, Paris, France

AP-HP, Pitié-Salpêtrière Hospital, Epilepsy Unit and Reference Center for Rare Epilepsies, F-75013, Paris, France Vincent Navarro Sorbonne Université, Institut du Cerveau - Paris Brain Institute - ICM, Inserm, CNRS, APHP, Pitié-Salpêtrière Hospital, Paris, France

Correspondence: vincent.navarro@aphp.fr
