# Supplementary material for: Sleep increases firing rate modulation during interictal epileptiform discharges in mesial temporal structures
Source: Brain Commun. 2026 Apr 30;8(3):fcag130. doi: 10.1093/braincomms/fcag130 (PMC13231451; doi:10.1093/braincomms/fcag130)
Supplement: fcag130_Supplementary_Data [file fcag130_supplementary_data.pdf]

Supplementary Materials

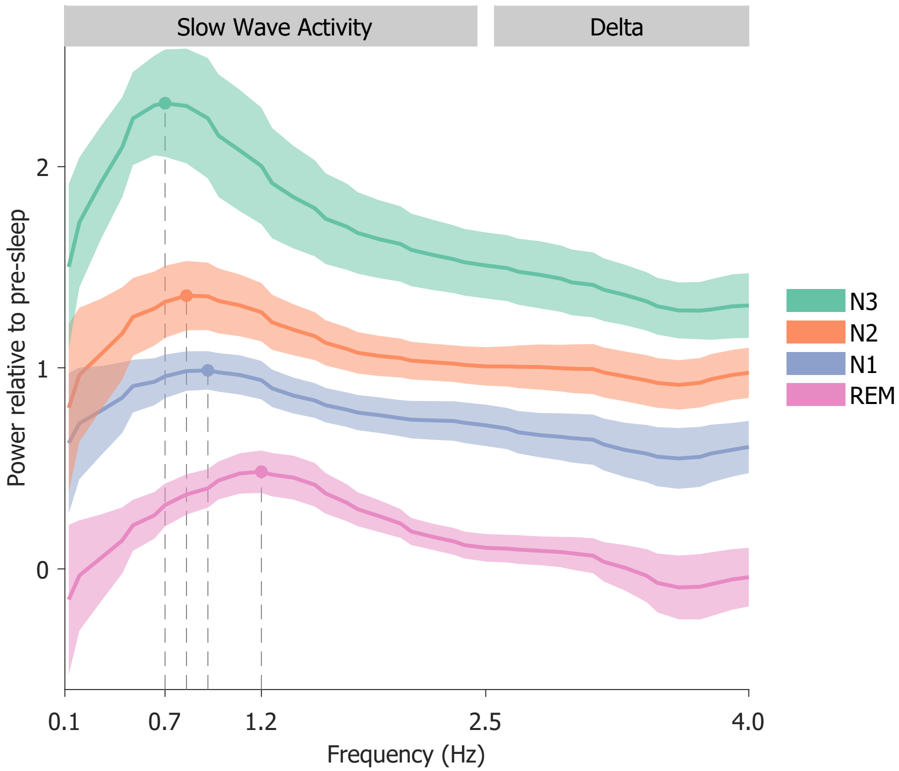

Supplementary Figure 1. Power in the Slow Wave Activity (SWA) and delta range, relative to the pre-sleep period, and separated per sleep stage. Lines show average power over patients per sleep stage, with shaded regions showing the standard error of the mean (N=8). Relative power showed a peak in the SWA frequency range ($0.1\,\mathrm{Hz}$ to $2.5\,\mathrm{Hz}$) which increased from $0.7\,\mathrm{Hz}$ in S3, to $0.8\,\mathrm{Hz}$ in S2, $0.9\,\mathrm{Hz}$ in S1, and $1.2\,\mathrm{Hz}$ in REM sleep.

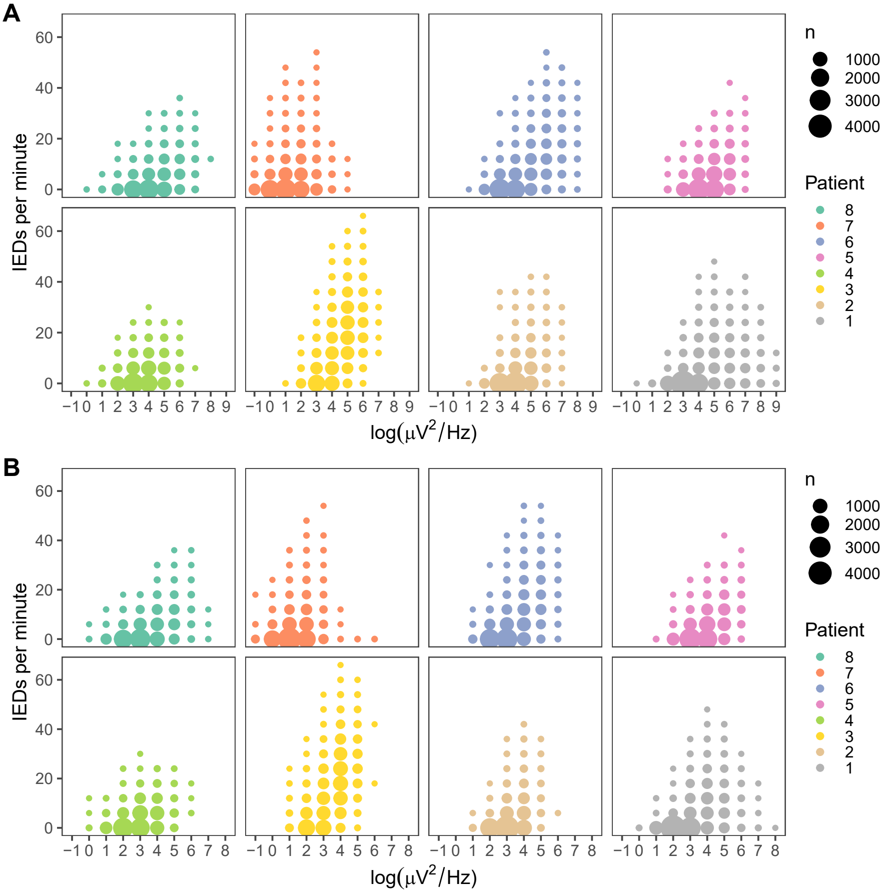

Supplementary Figure 2. Rates of interical epileptic discharges (IEDs) increase with increasing (**A**) slow wave activity ($0.1\,\mathrm{Hz}$ to $2.5\,\mathrm{Hz}$), and (**B**) delta ($2.5\,\mathrm{Hz}$ to $4\,\mathrm{Hz}$) power. For visualization, power was log-transformed, and both IED rates and power were binned. The size of the dots are proportional to the total number of observations in each bin. See Supplementary Table 7 for statistical test.

Supplementary Table 1. Anatomical locations of macro and micro electrodes

Patient	Side	Region	Subregion	SOZ
**Macro contacts**				
1	R	Hippocampal formation	Head of the Hippocampus	No
2	L	Hippocampal formation	Head of the Hippocampus	Yes
	L	Hippocampal formation	Hippocampus/CA1	Yes
3	L	Hippocampal formation	Head of the Hippocampus	Yes
	L	Hippocampal formation	Entorhinal cortex	No
4	L	Hippocampal formation	Hippocampus	No
	L	Hippocampal formation	Hippocampus/CA3	No
5	R	Hippocampal formation	Subiculum	Yes
	R	Nodule	Nodule	Yes
6	L	Hippocampal formation	Entorhinal cortex	No
	L	Hippocampal formation	Parahippocampus	No
7	R	Hippocampal formation	Entorhinal cortex	Yes
	R	Hippocampal formation	Hippocampus/CA1	Yes
8	R	Hippocampal formation	Hippocampus/CA1	Yes
**Micro electrodes**				
1	R	Hippocampal formation	Entorhinal cortex	Yes
2	L	Hippocampal formation	Entorhinal cortex	Yes
3	R	Temporo-basal cortex	Parahippocampus	No
	L	Temporo-basal cortex	Parahippocampus	Yes
4	L	Temporo-anterior lobe	Amygdala	No
	L	Mesial temporal cortex	CA3-CA4	No
5	R	Periventricular nodule	Nodule	Yes
	R	Periventricular nodule	Nodule	Yes
	R	Hippocampal formation	Subiculum	No
6	L	Hippocampal formation	Parahippocampal gyrus	No
7	R	Temporo-anterior lobe	Amygdala	Yes
8	R	Hippocampal formation	CA1/WM	Yes
	R	Hippocampal formation	CA1	Yes

Supplementary Table 2. Automatic IED detection performance

	Visual	Automatic detection				
Patient	24 hrs.	24 hrs.	Hit (%)	FA (%)	Total	Total hrs.
1	2106	3500	99.8	5.4	63345	453.5
2	3330	4618	99.3	6.9	9224	358.3
3	12912	12811	98.8	1.2	113832	305.6
4	3669	3664	97.9	11.0	43757	308.8
5	7086	4630	96.5	8.6	32782	237.2
6	3077	3054	98.9	3.8	29810	233.6
7	10439	3985	98.4	0.8	55465	305.6
8	2714	1965	94.1	14.3	53951	307.7
Mean	5667	4778	98.0	6.5	50271	313.8
Std.	4050	3359	1.9	4.7	30945	69.7

Supplementary Table 3. Anatomical locations of micro electrodes

Patient	Electrode	Contact	X	Y	Z
1	HaT2	1	27.0	-12.2	-27.1
		2	30.3	-12.9	-25.9
		3	36.9	-13.4	-24.7
		4	42.6	-14.1	-22.6
		5	49.3	-15.1	-22.3
2	HmT2	1	-18.8	-31.7	-13.5
		2	-23.8	-32.3	-11.9
		3	-28.8	-32.8	-11.5
		4	-33.9	-33.2	-11.0
		5	-39.1	-33.8	-10.6
2	HaT1	1	-20.6	-7.2	-30.3
		2	-23.4	-7.2	-27.7
		3	-28.2	-8.7	-24.6
		4	-34.0	-10.4	-21.4
3	Hm2g	1	-21.7	-30.9	-13.7
		2	-27.0	-32.8	-12.6
		3	-32.4	-35.2	-12.6
		4	-36.7	-37.5	-12.6
		5	-42.1	-39.9	-12.8
4	HmT2	1	-30.3	-25.4	-12.0
		2	-32.3	-27.2	-9.9
		3	-36.6	-31.8	-9.2
		4	-40.9	-36.1	-7.5
		5	-46.0	-41.4	-5.3
4	HaT2	1	-20.3	-9.0	-26.3
		2	-23.5	-10.2	-25.6
		3	-28.9	-10.9	-23.5
		4	-35.3	-12.8	-21.0
		5	-42.0	-13.8	-20.0
5	HmT3	1	30.0	-22.2	-17.5
		2	33.4	-22.3	-18.0
		3	40.3	-21.4	-19.1
		4	47.3	-21.4	-19.6
		5	54.3	-21.6	-20.2
5	HpNI	1	20.6	-32.7	-17.1
		2	25.3	-32.7	-15.5
		3	29.7	-32.5	-13.8
		4	35.1	-32.3	-11.3
		5	39.3	-32.0	-9.4
6	HaT2	1	-20.6	-10.5	-31.3
		2	-23.6	-10.5	-31.2
		3	-29.5	-10.6	-30.9
		4	-35.4	-10.5	-30.3
		5	-41.3	-10.3	-29.5

Continued on next page

Supplementary Table 3. Anatomical locations of micro electrodes (*continued*)

Patient	Electrode	Contact	X	Y	Z
6	HmT2	1	-26.9	-27.1	-21.5
		2	-29.8	-27.1	-21.3
		3	-35.6	-26.2	-22.4
		4	-41.6	-26.2	-22.9
		5	-47.5	-25.1	-22.2
7	HaTB	1	-18.7	-10.2	-25.9
		2	-20.9	-9.4	-26.2
		3	-27.5	-8.2	-27.6
		4	-32.9	-6.5	-28.0
		5	-39.8	-5.5	-29.6
8	HmT2	1	26.9	-20.4	-19.2
		2	29.9	-21.3	-18.5
		3	35.8	-23.1	-17.3
		4	40.8	-24.9	-15.6
8	HaT2	1	29.1	-16.2	-23.0
		2	32.1	-15.7	-21.9
		3	38.0	-14.8	-21.9
		4	43.6	-13.4	-20.6

Supplementary Table 4. Circular statistics of circadian epileptic activity shown in Figure 4

	Interictal activity			Seizures		
Patient	Time	Rayleigh	*p*	Time	Rayleigh	*p*
1	01:56	0.61	$<.0001^{***}$	17:43	0.76	$<.0001^{***}$
2	00:46	0.29	$<.0001^{***}$	08:56	0.36	0.55
3	01:37	0.50	$<.0001^{***}$	10:31	0.48	$<.01^{*}$
4	02:23	0.26	$<.0001^{***}$	18:26	0.94	$<.001^{**}$
5	01:48	0.28	$<.0001^{***}$	20:21	0.13	0.3
6	01:42	0.56	$<.0001^{***}$	03:33	0.57	0.14
7	17:25	0.08	$<.0001^{***}$	01:59	0.81	$<.0001^{***}$
8	03:38	0.41	$<.0001^{***}$	11:44	0.70	0.14

Circular Rayleigh Test: $. = p < 0.05$, $* = p < 0.01$, $** = p < 0.001$, $*** = p < 0.0001$

Supplementary Table 5. Time spend in sleep stages.

			% TST				
Patient	Night	TST	N3	N2	N1	REM	WASO
1	1	11.9	13	44	21	22	1.6
	2	8.7	14	42	18	25	0.9
	3	7.5	22	42	17	19	3
2	1	8.5	26	52	7	15	2.3
	2	7.6	33	39	8	19	0.4
	3	8.1	24	52	8	16	0.4
3	1	9.2	13	45	26	16	1.7
	2	7.2	27	33	24	16	1.7
	3	7.2	28	33	27	12	1.5
4	1	7.5	23	38	26	13	1.7
	2	6.7	16	41	26	17	1.3
	3	6.5	22	27	19	31	0.9
5	1	9.6	9	67	9	15	1.3
	2	7.2	26	42	10	22	0.4
	3	7.2	15	46	9	30	0.2
6	1	7.5	15	43	25	17	1.2
	2	8.2	21	35	15	29	0.9
	3	8.9	18	38	18	26	1.3
7	1	6.9	31	41	14	14	0.4
	2	9.6	28	46	7	19	0.5
	3	10.3	35	37	8	20	0.9
8	1	7	16	54	10	19	0.4
	2	7.1	10	52	16	22	0.6
	3	6.8	15	50	20	15	3.7
Mean		8.0	20.8	43.3	16.2	19.5	1.2

TST = Total sleep time (hrs.), WASO = Wake after sleep onset (hrs.)

Supplementary Table 6. Mixed effects model of sleep stage on IEDs rate show in Figure 5

Predictor	Coef β	SE(β)	df	z	*p*
**Sleep stages**					
Intercept	0.26	0.23	7.03	1.12	0.3
N3	1.13	0.01	93176.95	79.21	$<.0001^{***}$
N2	0.90	0.01	93176.88	70.85	$< .0001^{***}$
N1	0.54	0.02	93176.31	35.04	$<.0001^{***}$
WASO	0.10	0.02	93176.64	6.08	$<.0001^{***}$
REM	0.00	0.01	93176.50	-0.31	0.76
Post	0.01	0.02	93176.92	0.43	0.67
**Post-hoc comparisons**					
Pre - N3	-1.13	0.01		-79.21	$<.0001^{***}$
Pre - N2	-0.90	0.01		-70.85	$<.0001^{***}$
Pre - N1	-0.54	0.02		-35.04	$<.0001^{***}$
Pre - WASO	-0.10	0.02		-6.08	$<.0001^{***}$
Pre - REM	0.00	0.01		0.31	1
Pre - Post	-0.01	0.02		-0.43	1
N3 - N2	0.23	0.01		21.19	$<.0001^{***}$
N3 - N1	0.59	0.01		41.55	$<.0001^{***}$
N3 - WASO	1.03	0.02		63.83	$<.0001^{***}$
N3 - REM	1.14	0.01		85.89	$<.0001^{***}$
N3 - Post	1.12	0.01		80.70	$<.0001^{***}$
N2 - N1	0.36	0.01		28.35	$<.0001^{***}$
N2 - WASO	0.79	0.01		54.16	$<.0001^{***}$
N2 - REM	0.90	0.01		78.83	$<.0001^{***}$
N2 - Post	0.89	0.01		72.45	$<.0001^{***}$
N1 - WASO	0.43	0.02		25.56	$<.0001^{***}$
N1 - REM	0.54	0.01		37.38	$<.0001^{***}$
N1 - Post	0.53	0.02		35.00	$<.0001^{***}$
WASO - REM	0.11	0.02		6.65	$<.0001^{***}$
WASO - Post	0.10	0.02		5.78	$<.0001^{***}$
REM - Post	-0.01	0.01		-0.77	0.99

$. = p < 0.05$, $* = p < 0.01$, $** = p < 0.001$, $*** = p < 0.0001$,
TST = Total sleep time (hrs.), WASO = Wake after sleep onset

Supplementary Table 7. Mixed effects model of Slow Wave activity (0.1-2.5Hz) and Delta power (2.5-4Hz) on rate of IEDs

	Coef β	SE(β)	df	z	*p*
Intercept	0.412	0.217	7	1.90	0.1
Slow Wave	0.000	0.000	93182	14.02	$<.0001^{***}$
Delta	0.010	0.000	93184	101.95	$<.0001^{***}$

$. = p < 0.05$, $* = p < 0.01$, $** = p < 0.001$, $*** = p < 0.0001$

Supplementary Table 8. Mixed effects model of sleep stage on Slow Wave (0.1-2.5Hz) power show in Figure 5

Predictor	Coef β	SE(β)	df	z	*p*
**Coefficients**					
Intercept	29.03	25.16	7.11	1.15	0.29
N3	264.51	3.00	93179.16	88.30	<.0001***
N2	93.86	2.65	93178.96	35.37	<.0001***
N1	48.34	3.22	93177.11	15.00	<.0001***
WASO	18.02	3.59	93178.22	5.02	<.0001***
REM	14.50	3.05	93177.76	4.76	<.0001***
Post	5.69	3.20	93179.09	1.78	0.08
**Post-hoc comparisons**					
Pre - N3	-264.51	3.00		-88.30	<.0001***
Pre - N2	-93.86	2.65		-35.37	<.0001***
Pre - N1	-48.34	3.22		-15.00	<.0001***
Pre - WASO	-18.02	3.59		-5.02	<.0001***
Pre - REM	-14.50	3.05		-4.76	<.0001***
Pre - Post	-5.69	3.20		-1.78	0.56
N3 - N2	170.65	2.33		73.38	<.0001***
N3 - N1	216.17	2.99		72.20	<.0001***
N3 - WASO	246.49	3.38		73.03	<.0001***
N3 - REM	250.02	2.77		90.14	<.0001***
N3 - Post	258.82	2.92		88.53	<.0001***
N2 - N1	45.52	2.65		17.17	<.0001***
N2 - WASO	75.84	3.07		24.72	<.0001***
N2 - REM	79.36	2.40		33.11	<.0001***
N2 - Post	88.17	2.58		34.22	<.0001***
N1 - WASO	30.32	3.56		8.51	<.0001***
N1 - REM	33.85	3.04		11.12	<.0001***
N1 - Post	42.65	3.19		13.39	<.0001***
WASO - REM	3.52	3.42		1.03	0.95
WASO - Post	12.33	3.54		3.48	<.01*
REM - Post	8.81	2.99		2.95	<.05

$. = p < 0.05$, $* = p < 0.01$, $** = p < 0.001$, $*** = p < 0.0001$, TST = Total sleep time (hrs.), WASO = Wake after sleep onset

Supplementary Table 9. Mixed effects model of sleep stage on Delta (2.5-4 Hz) power show in Figure 5

Predictor	Coef β	SE(β)	df	z	*p*
**Coefficients**					
Intercept	15.24	6.58	7.05	2.31	0.05
N3	54.64	0.55	93177.66	99.54	$<.0001^{***}$
N2	29.96	0.49	93177.55	61.61	$<.0001^{***}$
N1	16.54	0.59	93176.55	28.02	$<.0001^{***}$
WASO	3.96	0.66	93177.13	6.02	$<.0001^{***}$
REM	2.25	0.56	93176.89	4.03	$<.0001^{***}$
Post	0.22	0.59	93177.62	0.38	0.71
**Post-hoc comparisons**					
Pre - N3	-54.64	0.55		-99.54	$<.0001^{***}$
Pre - N2	-29.96	0.49		-61.61	$<.0001^{***}$
Pre - N1	-16.54	0.59		-28.02	$<.0001^{***}$
Pre - WASO	-3.96	0.66		-6.02	$<.0001^{***}$
Pre - REM	-2.25	0.56		-4.03	$<.01^{*}$
Pre - Post	-0.22	0.59		-0.38	1
N3 - N2	24.68	0.43		57.92	$<.0001^{***}$
N3 - N1	38.09	0.55		69.44	$<.0001^{***}$
N3 - WASO	50.68	0.62		81.95	$<.0001^{***}$
N3 - REM	52.39	0.51		103.08	$<.0001^{***}$
N3 - Post	54.42	0.54		101.58	$<.0001^{***}$
N2 - N1	13.42	0.49		27.63	$<.0001^{***}$
N2 - WASO	26.00	0.56		46.25	$<.0001^{***}$
N2 - REM	27.71	0.44		63.09	$<.0001^{***}$
N2 - Post	29.74	0.47		63.00	$<.0001^{***}$
N1 - WASO	12.59	0.65		19.28	$<.0001^{***}$
N1 - REM	14.30	0.56		25.62	$<.0001^{***}$
N1 - Post	16.32	0.58		27.96	$<.0001^{***}$
WASO - REM	1.71	0.63		2.73	0.09
WASO - Post	3.74	0.65		5.76	$<.0001^{***}$
REM - Post	2.03	0.55		3.70	$<.01^{*}$

$. = p < 0.05$, $* = p < 0.01$, $** = p < 0.001$, $*** = p < 0.0001$,
TST = Total sleep time (hrs.), WASO = Wake after sleep onset

Supplementary Table 10. Number of responsive or unresponsive putatively isolated single units (SUA) and multiunits (MUA)

	SUA		MUA	
Patient	Responsive	Unresponsive	Responsive	Unresponsive
1	47	4	36	3
2	8	0	2	0
3	1	5	5	19
4	2	0	15	0
5	4	10	6	13
6	5	1	10	0
7	0	6	3	6
8	18	1	9	4
Sum	85	27	86	45
Responsive (%)	75.9	24.1	65.6	34.4

Supplementary Table 11. Mixed effects model of sleep stage on Event Related Potential (ERP) peak amplitude of interical epileptic discharge (IED) shown in Figure 7

	Spike amplitude					Slow wave amplitude					Difference amplitude				
	Coef β	SE(β)	df	z	p	Coef β	SE(β)	df	z	p	Coef β	SE(β)	df	z	p
**Sleep stages**															
Intercept	425.78	108.40	7	3.93	$<.01^{*}$	-260.53	97.66	7	-2.67	$<.05^{\cdot}$	-260.53	97.66	7	-2.67	$<.05^{\cdot}$
N3	84.05	2.98	78969	28.19	$<.0001^{***}$	-29.52	4.24	78968	-6.96	$<.0001^{***}$	-29.52	4.24	78968	-6.96	$<.0001^{***}$
N2	63.59	2.83	78967	22.46	$<.0001^{***}$	-35.60	4.03	78967	-8.85	$<.0001^{***}$	-35.60	4.03	78967	-8.85	$<.0001^{***}$
N1	49.95	3.18	78966	15.70	$<.0001^{***}$	-29.32	4.53	78966	-6.48	$<.0001^{***}$	-29.32	4.53	78966	-6.48	$<.0001^{***}$
WASO	19.01	3.88	78965	4.91	$<.0001^{***}$	-10.38	5.51	78966	-1.88	0.06	-10.38	5.51	78966	-1.88	0.06
Post	7.56	4.35	78970	1.74	0.08	5.74	6.18	78969	0.93	0.35	5.74	6.18	78969	0.93	0.35
REM	-61.68	4.33	78977	-14.24	$<.0001^{***}$	87.42	6.16	78973	14.19	$<.0001^{***}$	87.42	6.16	78973	14.19	$<.0001^{***}$
**Post-hoc comparisons**															
Pre - N3	-84.05	2.98		-28.19	$<.0001^{***}$	29.52	4.24		6.96	$<.0001^{***}$	-113.58	5.45		-20.82	$<.0001^{***}$
Pre - N2	-63.59	2.83		-22.46	$<.0001^{***}$	35.60	4.03		8.85	$<.0001^{***}$	-99.18	5.18		-19.15	$<.0001^{***}$
Pre - N1	-49.95	3.18		-15.70	$<.0001^{***}$	29.32	4.53		6.48	$<.0001^{***}$	-79.26	5.82		-13.61	$<.0001^{***}$
Pre - WASO	-19.01	3.88		-4.91	$<.0001^{***}$	10.38	5.51		1.88	0.49	-29.38	7.09		-4.14	$<.001^{**}$
Pre - REM	-7.56	4.35		-1.74	0.59	-5.74	6.18		-0.93	0.97	-1.78	7.95		-0.22	1
Pre - Post	61.68	4.33		14.24	$<.0001^{***}$	-87.42	6.16		-14.19	$<.0001^{***}$	149.09	7.92		18.81	$<.0001^{***}$
N3 - N2	20.46	1.76		11.63	$<.0001^{***}$	6.08	2.50		2.43	0.19	14.40	3.22		4.47	$<.001^{**}$
N3 - N1	34.10	2.38		14.32	$<.0001^{***}$	-0.21	3.39		-0.06	1	34.32	4.36		7.88	$<.0001^{***}$
N3 - WASO	65.04	3.26		19.95	$<.0001^{***}$	-19.14	4.63		-4.13	$<.001^{**}$	84.19	5.96		14.12	$<.0001^{***}$
N3 - REM	76.49	3.76		20.32	$<.0001^{***}$	-35.26	5.35		-6.59	$<.0001^{***}$	111.80	6.89		16.23	$<.0001^{***}$
N3 - Post	145.73	3.80		38.33	$<.0001^{***}$	-116.94	5.41		-21.63	$<.0001^{***}$	262.67	6.95		37.77	$<.0001^{***}$
N2 - N1	13.64	2.17		6.30	$<.0001^{***}$	-6.29	3.08		-2.04	0.39	19.92	3.96		5.03	$<.0001^{***}$
N2 - WASO	44.58	3.12		14.29	$<.0001^{***}$	-25.22	4.44		-5.68	$<.0001^{***}$	69.80	5.71		12.23	$<.0001^{***}$
N2 - REM	56.03	3.66		15.32	$<.0001^{***}$	-41.34	5.20		-7.95	$<.0001^{***}$	97.40	6.69		14.55	$<.0001^{***}$
N2 - Post	125.27	3.69		33.91	$<.0001^{***}$	-123.02	5.25		-23.42	$<.0001^{***}$	248.27	6.76		36.73	$<.0001^{***}$
N1 - WASO	30.94	3.44		9.00	$<.0001^{***}$	-18.93	4.89		-3.87	$<.01^{*}$	49.87	6.29		7.93	$<.0001^{***}$
N1 - REM	42.39	3.95		10.72	$<.0001^{***}$	-35.05	5.62		-6.23	$<.0001^{***}$	77.48	7.23		10.71	$<.0001^{***}$
N1 - Post	111.63	4.01		27.85	$<.0001^{***}$	-116.73	5.70		-20.48	$<.0001^{***}$	228.35	7.33		31.15	$<.0001^{***}$
WASO - REM	11.45	4.55		2.52	0.15	-16.12	6.46		-2.49	0.16	27.61	8.32		3.32	$<.05^{\cdot}$
WASO - Post	80.69	4.59		17.56	$<.0001^{***}$	-97.80	6.53		-14.97	$<.0001^{***}$	178.48	8.41		21.23	$<.0001^{***}$
REM - Post	69.23	4.95		13.98	$<.0001^{***}$	-81.68	7.04		-11.59	$<.0001^{***}$	150.87	9.06		16.65	$<.0001^{***}$

$. = p < 0.05$, $* = p < 0.01$, $** = p < 0.001$, $*** = p < 0.0001$

Supplementary Table 12. Mixed effects model of sleep stage on spontaneous neuronal behaviour shown in Figure 10 & Figure 11

	Firing Rate (SUA)					Firing Rate (MUA)					Bursts					Amplitude					CV2				
	Coef β	SE(β)	df	z	p	Coef β	SE(β)	df	z	p	Coef β	SE(β)	df	z	p	Coef β	SE(β)	df	z	p	Coef β	SE(β)	df	z	p
**Sleep stages**																									
Intercept	7.12	0.33	123	21.62	<.0001***	10.80	1.11	6	9.70	<.0001***	23.18	4.19	6	5.53	<.01*	67.65	11.55	8	5.86	<.001**	0.92	0.01	2	77.06	<.001**
N3	-3.85	0.07	28868	-54.95	<.0001***	-5.95	0.11	78794	-53.18	<.0001***	-16.91	0.26	107669	-64.55	<.0001***	0.46	0.05	107584	8.78	<.0001***	0.11	0.00	107596	87.57	<.0001***
N2	-3.07	0.06	28877	-55.06	<.0001***	-3.87	0.08	78811	-46.14	<.0001***	-12.13	0.20	107692	-61.12	<.0001***	0.29	0.04	107584	7.28	<.0001***	0.07	0.00	107611	75.09	<.0001***
N1	-2.77	0.06	28866	-43.48	<.0001***	-3.29	0.10	78799	-33.20	<.0001***	-9.95	0.23	107668	-42.69	<.0001***	0.21	0.05	107584	4.49	<.0001***	0.06	0.00	107595	49.32	<.0001***
WASO	-2.57	0.07	28880	-36.80	<.0001***	-2.59	0.10	78827	-25.89	<.0001***	-8.67	0.24	107704	-35.86	<.0001***	0.59	0.05	107585	12.23	<.0001***	0.04	0.00	107588	33.40	<.0001***
REM	-2.02	0.06	28873	-34.64	<.0001***	-2.64	0.08	78801	-31.05	<.0001***	-9.20	0.20	107680	-45.27	<.0001***	0.20	0.04	107584	4.93	<.0001***	0.03	0.00	107604	32.59	<.0001***
Post	-1.97	0.06	28875	-30.98	<.0001***	0.26	0.09	78824	2.82	<.01*	-5.82	0.22	107699	-26.61	<.0001***	-0.19	0.04	107584	-4.23	<.0001***	0.01	0.00	107618	12.42	<.0001***
**Post-hoc comparisons**																									
Pre - N3	3.85	0.07		54.95	<.0001***	5.95	0.11		53.18	<.0001***	16.91	0.26		64.55	<.0001***	-0.46	0.05		-8.78	<.0001***	-0.11	0.00		-87.57	<.0001***
Pre - N2	3.07	0.06		55.06	<.0001***	3.87	0.08		46.14	<.0001***	12.13	0.20		61.12	<.0001***	-0.29	0.04		-7.28	<.0001***	-0.07	0.00		-75.09	<.0001***
Pre - N1	2.77	0.06		43.48	<.0001***	3.29	0.10		33.20	<.0001***	9.95	0.23		42.69	<.0001***	-0.21	0.05		-4.49	<.001**	-0.06	0.00		-49.32	<.0001***
Pre - WASO	2.57	0.07		36.80	<.0001***	2.59	0.10		25.89	<.0001***	8.67	0.24		35.86	<.0001***	-0.59	0.05		-12.23	<.0001***	-0.04	0.00		-33.40	<.0001***
Pre - REM	2.02	0.06		34.64	<.0001***	2.64	0.08		31.05	<.0001***	9.20	0.20		45.27	<.0001***	-0.20	0.04		-4.93	<.0001***	-0.03	0.00		-32.59	<.0001***
Pre - Post	1.97	0.06		30.98	<.0001***	-0.26	0.09		-2.82	0.07	5.82	0.22		26.61	<.0001***	0.19	0.04		4.23	<.001**	-0.01	0.00		-12.42	<.0001***
N3 - N2	-0.78	0.06		-13.80	<.0001***	-2.08	0.11		-19.36	<.0001***	-4.79	0.24		-19.73	<.0001***	0.17	0.05		3.53	<.01*	0.04	0.00		33.17	<.0001***
N3 - N1	-1.08	0.07		-16.48	<.0001***	-2.67	0.12		-21.84	<.0001***	-6.97	0.28		-25.15	<.0001***	0.25	0.06		4.53	<.001**	0.06	0.00		41.36	<.0001***
N3 - WASO	-1.28	0.07		-17.61	<.0001***	-3.36	0.12		-26.90	<.0001***	-8.25	0.29		-28.50	<.0001***	-0.13	0.06		-2.27	0.26	0.07	0.00		51.39	<.0001***
N3 - REM	-1.84	0.06		-30.89	<.0001***	-3.32	0.11		-30.05	<.0001***	-7.72	0.25		-30.77	<.0001***	0.26	0.05		5.18	<.0001***	0.08	0.00		65.09	<.0001***
N3 - Post	-1.88	0.07		-28.68	<.0001***	-6.21	0.12		-53.95	<.0001***	-11.10	0.27		-41.83	<.0001***	0.65	0.05		12.16	<.0001***	0.10	0.00		76.26	<.0001***
N2 - N1	-0.30	0.05		-5.93	<.0001***	-0.58	0.10		-5.97	<.0001***	-2.18	0.22		-9.96	<.0001***	0.08	0.04		1.82	0.53	0.02	0.00		15.58	<.0001***
N2 - WASO	-0.50	0.06		-8.45	<.0001***	-1.27	0.10		-12.68	<.0001***	-3.46	0.23		-14.81	<.0001***	-0.30	0.05		-6.47	<.0001***	0.03	0.00		29.21	<.0001***
N2 - REM	-1.06	0.04		-24.74	<.0001***	-1.23	0.08		-14.95	<.0001***	-2.93	0.19		-15.84	<.0001***	0.09	0.04		2.39	0.2	0.04	0.00		44.74	<.0001***
N2 - Post	-1.10	0.05		-22.00	<.0001***	-4.12	0.09		-46.57	<.0001***	-6.31	0.20		-31.02	<.0001***	0.47	0.04		11.65	<.0001***	0.06	0.00		59.91	<.0001***
N1 - WASO	-0.21	0.07		-3.06	<.05.	-0.69	0.11		-6.13	<.0001***	-1.28	0.26		-4.88	<.0001***	-0.38	0.05		-7.26	<.0001***	0.02	0.00		13.00	<.0001***
N1 - REM	-0.76	0.05		-14.34	<.0001***	-0.65	0.10		-6.58	<.0001***	-0.75	0.22		-3.36	<.05.	0.01	0.04		0.19	1	0.02	0.00		21.73	<.0001***
N1 - Post	-0.80	0.06		-13.80	<.0001***	-3.54	0.10		-34.21	<.0001***	-4.13	0.24		-17.38	<.0001***	0.39	0.05		8.29	<.0001***	0.04	0.00		36.93	<.0001***
WASO - REM	-0.55	0.06		-8.90	<.0001***	0.04	0.10		0.42	1	0.53	0.24		2.22	0.28	0.39	0.05		8.18	<.0001***	0.01	0.00		6.08	<.0001***
WASO - Post	-0.60	0.07		-9.00	<.0001***	-2.85	0.11		-26.88	<.0001***	-2.85	0.25		-11.37	<.0001***	0.78	0.05		15.50	<.0001***	0.03	0.00		21.39	<.0001***
REM - Post	-0.04	0.05		-0.80	0.99	-2.89	0.09		-32.10	<.0001***	-3.38	0.21		-16.13	<.0001***	0.39	0.04		9.20	<.0001***	0.02	0.00		18.64	<.0001***

$. = p < 0.05, * = p < 0.01, ** = p < 0.001, *** = p < 0.0001$

Supplementary Table 13. Effect of sleep stage on firing rates during IEDs shown in Figure 8 & Figure 9

	Spike					Wave					Ratio				
	Coef β	SE(β)	df	z	p	Coef β	SE(β)	df	z	p	Coef β	SE(β)	df	z	p
**Sleep stages**															
Intercept	6.20	1.49	6	4.17	<.01*	11.05	3.08	7	3.59	<.01*	4.83	2.20	7	2.19	0.07
N3	-2.21	0.38	1237	-5.88	<.0001***	2.66	0.65	1237	4.12	<.0001***	4.87	0.58	1237	8.45	<.0001***
N2	-2.01	0.38	1237	-5.33	<.0001***	3.20	0.65	1237	4.95	<.0001***	5.20	0.58	1237	9.02	<.0001***
N1	-1.68	0.38	1237	-4.45	<.0001***	3.30	0.65	1237	5.11	<.0001***	4.98	0.58	1237	8.63	<.0001***
WASO	-2.03	0.38	1237	-5.38	<.0001***	1.34	0.65	1237	2.08	<.05.	3.37	0.58	1237	5.84	<.0001***
Post	-0.68	0.38	1237	-1.82	0.07	-0.19	0.65	1237	-0.29	0.77	0.50	0.58	1237	0.86	0.39
REM	-0.20	0.38	1237	-0.51	0.61	-1.28	0.65	1237	-1.97	<.05.	-1.09	0.58	1237	-1.87	0.06
**Post-hoc comparisons**															
Pre - N3	2.21	0.38		5.88	<.0001***	-2.66	0.65		-4.12	<.001**	-4.87	0.58		-8.45	<.0001***
Pre - N2	2.01	0.38		5.33	<.0001***	-3.20	0.65		-4.95	<.0001***	-5.20	0.58		-9.02	<.0001***
Pre - N1	1.68	0.38		4.45	<.001**	-3.30	0.65		-5.11	<.0001***	-4.98	0.58		-8.63	<.0001***
Pre - WASO	2.03	0.38		5.38	<.0001***	-1.34	0.65		-2.08	0.37	-3.37	0.58		-5.84	<.0001***
Pre - REM	0.68	0.38		1.82	0.54	0.19	0.65		0.29	1	-0.50	0.58		-0.86	0.98
Pre - Post	0.20	0.38		0.51	1	1.28	0.65		1.97	0.44	1.09	0.58		1.87	0.5
N3 - N2	-0.21	0.38		-0.55	1	-0.54	0.65		-0.83	0.98	-0.33	0.58		-0.57	1
N3 - N1	-0.54	0.38		-1.42	0.79	-0.64	0.65		-0.99	0.96	-0.11	0.58		-0.18	1
N3 - WASO	-0.19	0.38		-0.50	1	1.32	0.65		2.05	0.39	1.51	0.58		2.61	0.12
N3 - REM	-1.53	0.38		-4.06	<.01*	2.85	0.65		4.41	<.001**	4.38	0.58		7.59	<.0001***
N3 - Post	-2.02	0.38		-5.32	<.0001***	3.94	0.65		6.06	<.0001***	5.96	0.58		10.26	<.0001***
N2 - N1	-0.33	0.38		-0.87	0.98	-0.10	0.65		-0.16	1	0.22	0.58		0.39	1
N2 - WASO	0.02	0.38		0.05	1	1.86	0.65		2.88	0.06	1.84	0.58		3.18	<.05.
N2 - REM	-1.32	0.38		-3.51	<.01*	3.38	0.65		5.24	<.0001***	4.70	0.58		8.16	<.0001***
N2 - Post	-1.81	0.38		-4.77	<.0001***	4.48	0.65		6.89	<.0001***	6.29	0.58		10.83	<.0001***
N1 - WASO	0.35	0.38		0.93	0.97	1.96	0.65		3.04	<.05.	1.61	0.58		2.80	0.08
N1 - REM	-0.99	0.38		-2.64	0.12	3.49	0.65		5.40	<.0001***	4.48	0.58		7.77	<.0001***
N1 - Post	-1.48	0.38		-3.91	<.01*	4.58	0.65		7.05	<.0001***	6.07	0.58		10.44	<.0001***
WASO - REM	-1.34	0.38		-3.56	<.01*	1.53	0.65		2.36	0.21	2.87	0.58		4.97	<.0001***
WASO - Post	-1.83	0.38		-4.83	<.0001***	2.62	0.65		4.03	<.01*	4.45	0.58		7.67	<.0001***
REM - Post	-0.49	0.38		-1.29	0.86	1.09	0.65		1.68	0.63	1.58	0.58		2.73	0.09

$. = p < 0.05, * = p < 0.01, ** = p < 0.001, *** = p < 0.0001$

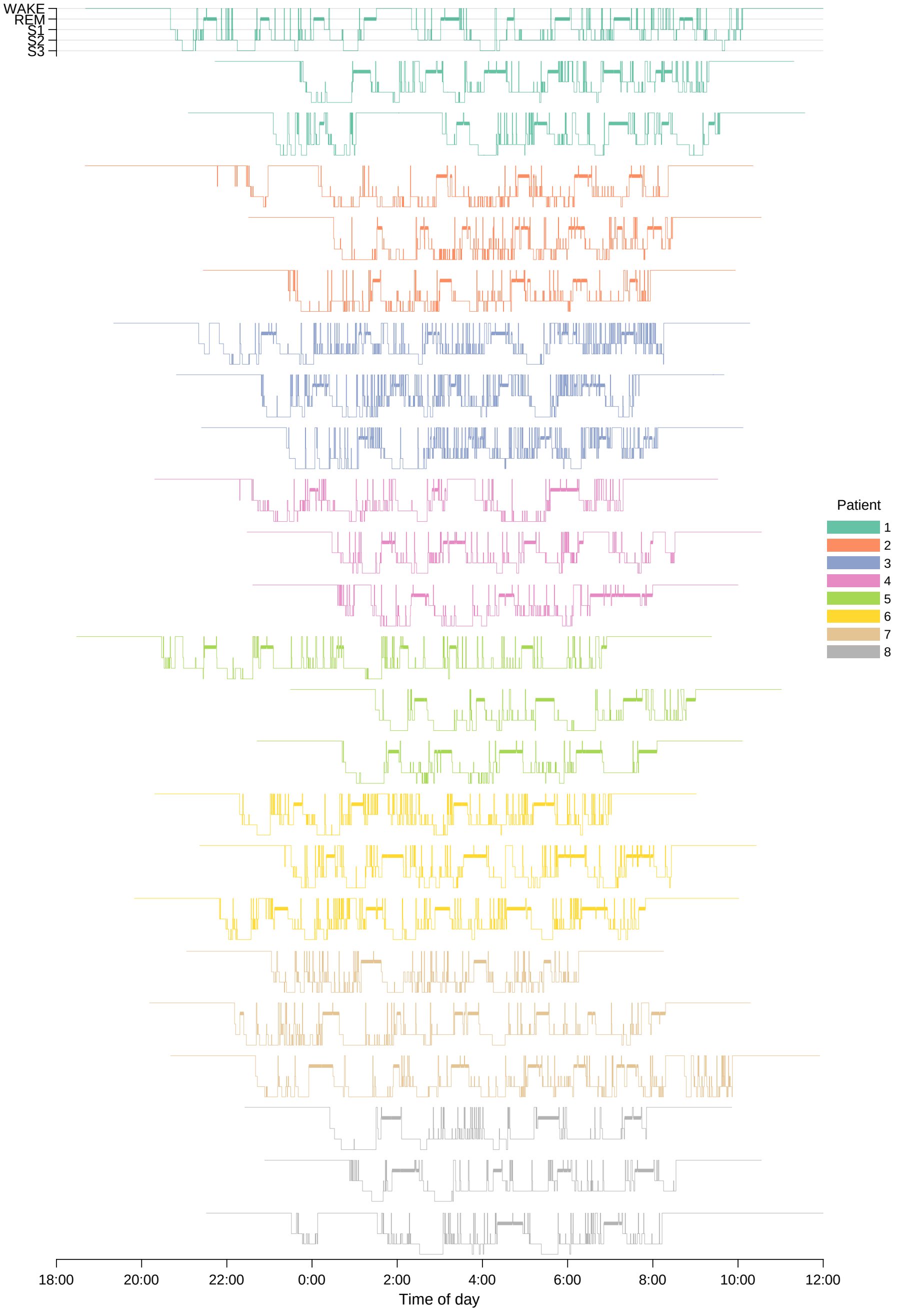

Supplementary Figure 3. Hypnograms of all 8 patients, including 2 hours pre-sleep and post-sleep.

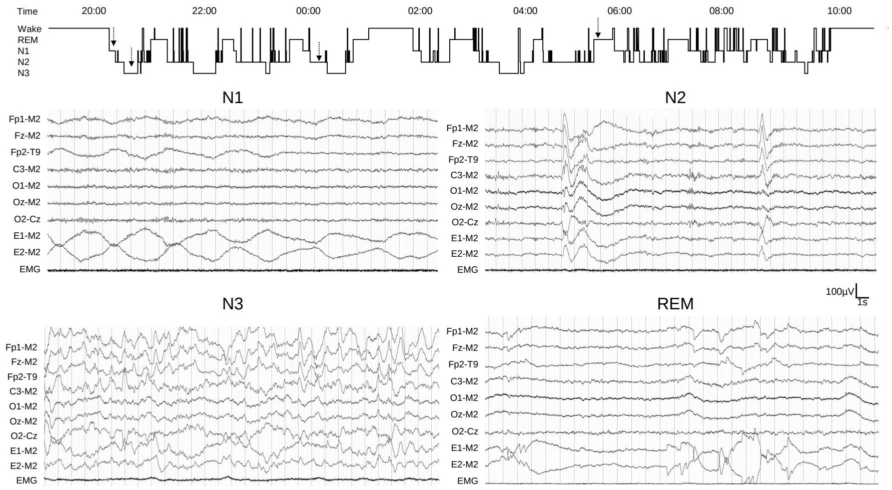

Supplementary Figure 4. Example of data used of sleep staging Patient nr. 1

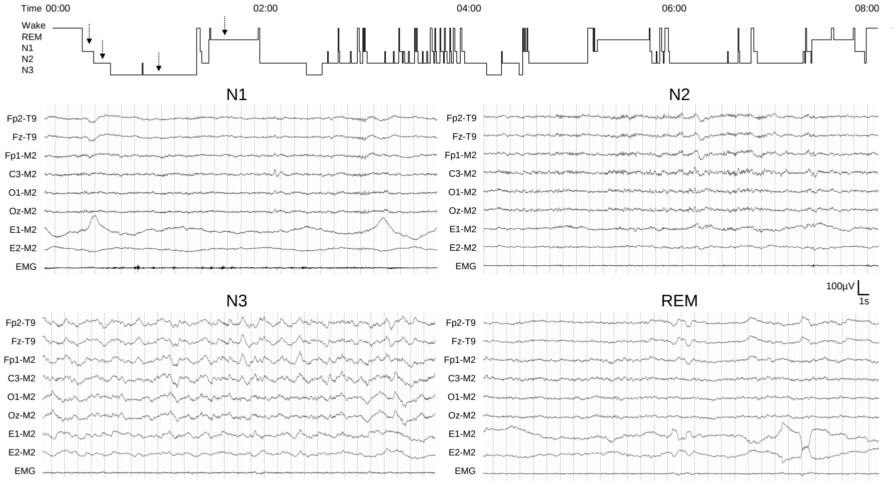

Supplementary Figure 5. Example of data used for sleep staging Patient nr. 8

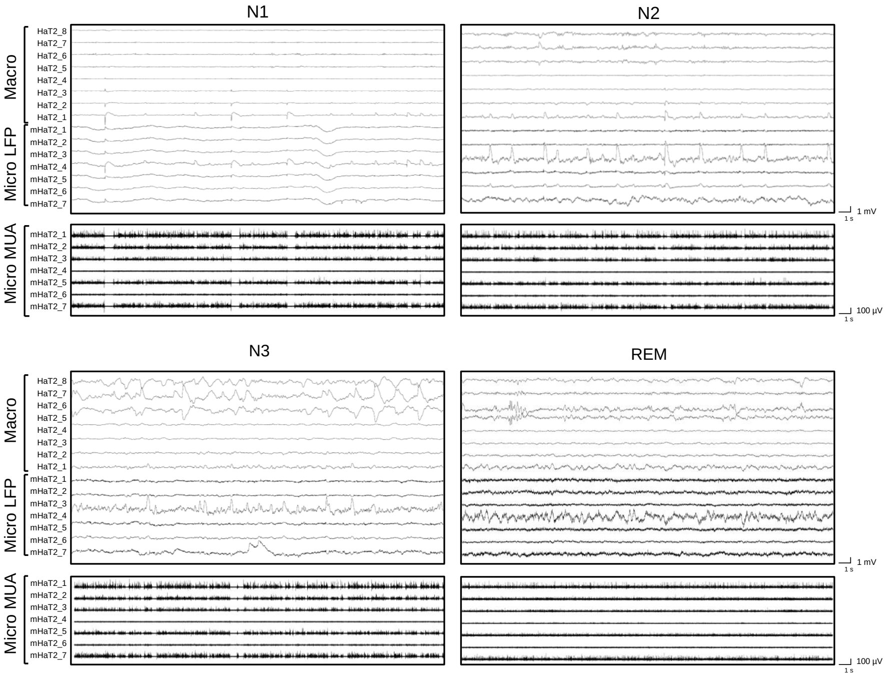

Supplementary Figure 6. Example of macro- and micro electrode recordings during sleep stages.

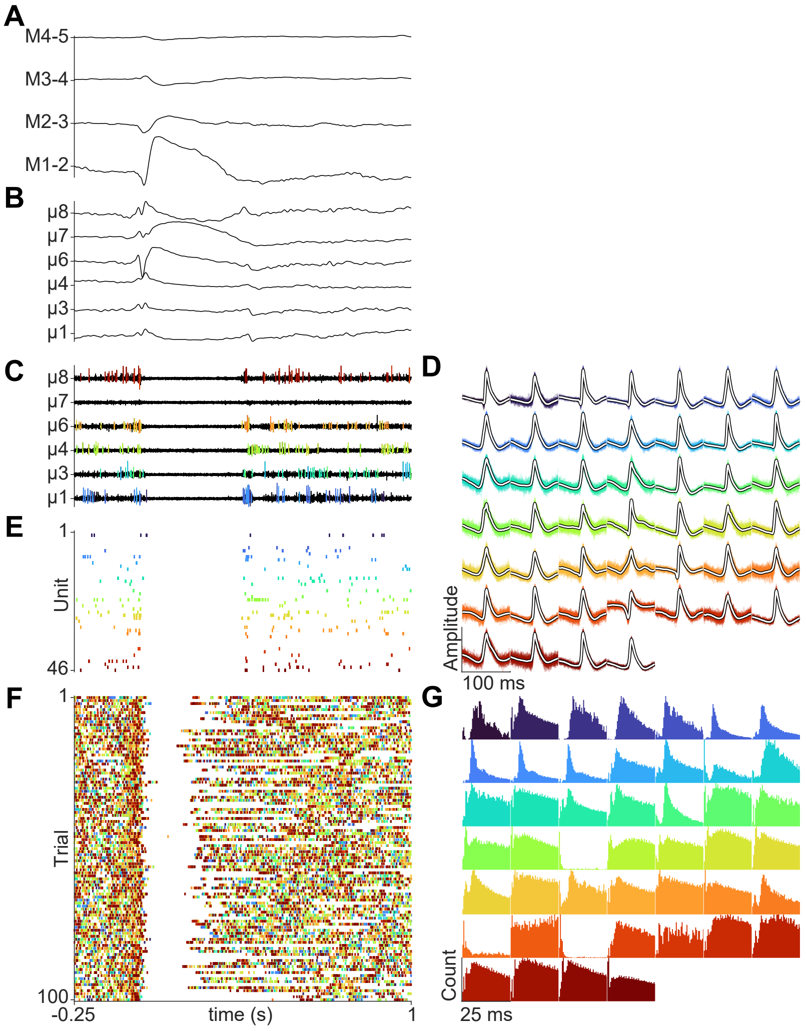

Supplementary Figure 7. Example of macro- and micro electrode signals timelocked to interictal epileptiform discharges at $-0.25\,\mathrm{s}$ to $1\,\mathrm{s}$ with respect to interictal epileptiform discharge (IED) peak, as well a unit descriptives in color consistent across panels. A) Low-pass filtered local field potentials (LFPs) on macroelectrode contacts in bipolar referencing indicated in the labels. B) High-pass filtered LFPs from micro electrode. C) micro electrode data showing multi-unit activity, with units indicated in color. D) Unit waveform averages overlaid on 100 example waveforms. E) Rasterplot of unit firing times per unit. Rasterplot of all units for the first 100 IEDs. G. Spike-time histograms.

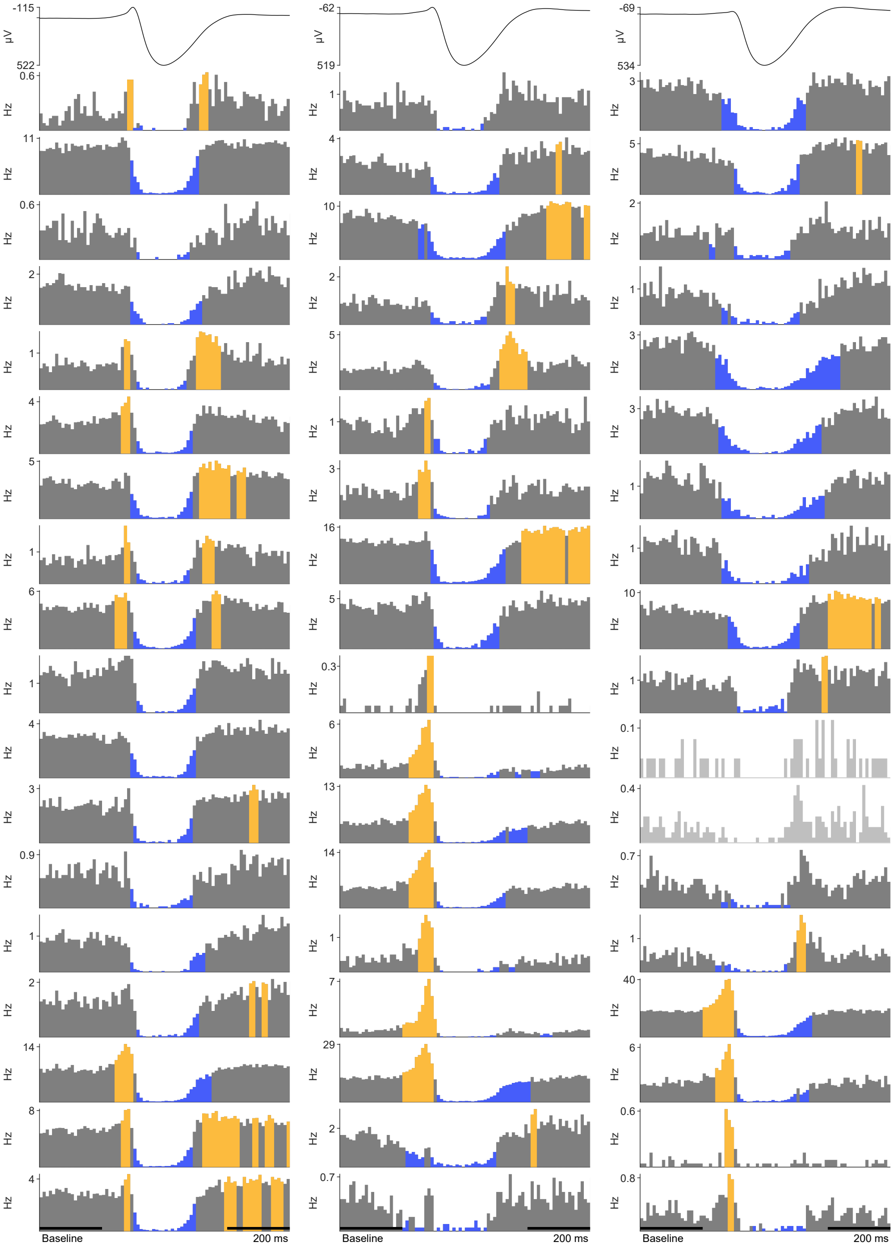

Supplementary Figure 8. Patient 1 (1/3): peri-stimulus time histogram of each unit. Top row shows time-locked local field potential (LFP). Units are organized over nights (columns). A threshold of $p < 0.01$ (first-level t-test) was used to determine contiguous temporal clusters, after which a threshold of $p < 0.05$ (one-sided correction) determined whether the clusters could be explained by permutation (sum of t values, $n = 10.000$) (1). The yellow color indicates significant increases and the blue color decreases compared with the average firing rate during baseline, indicated in the x-axis of bottom left plot.

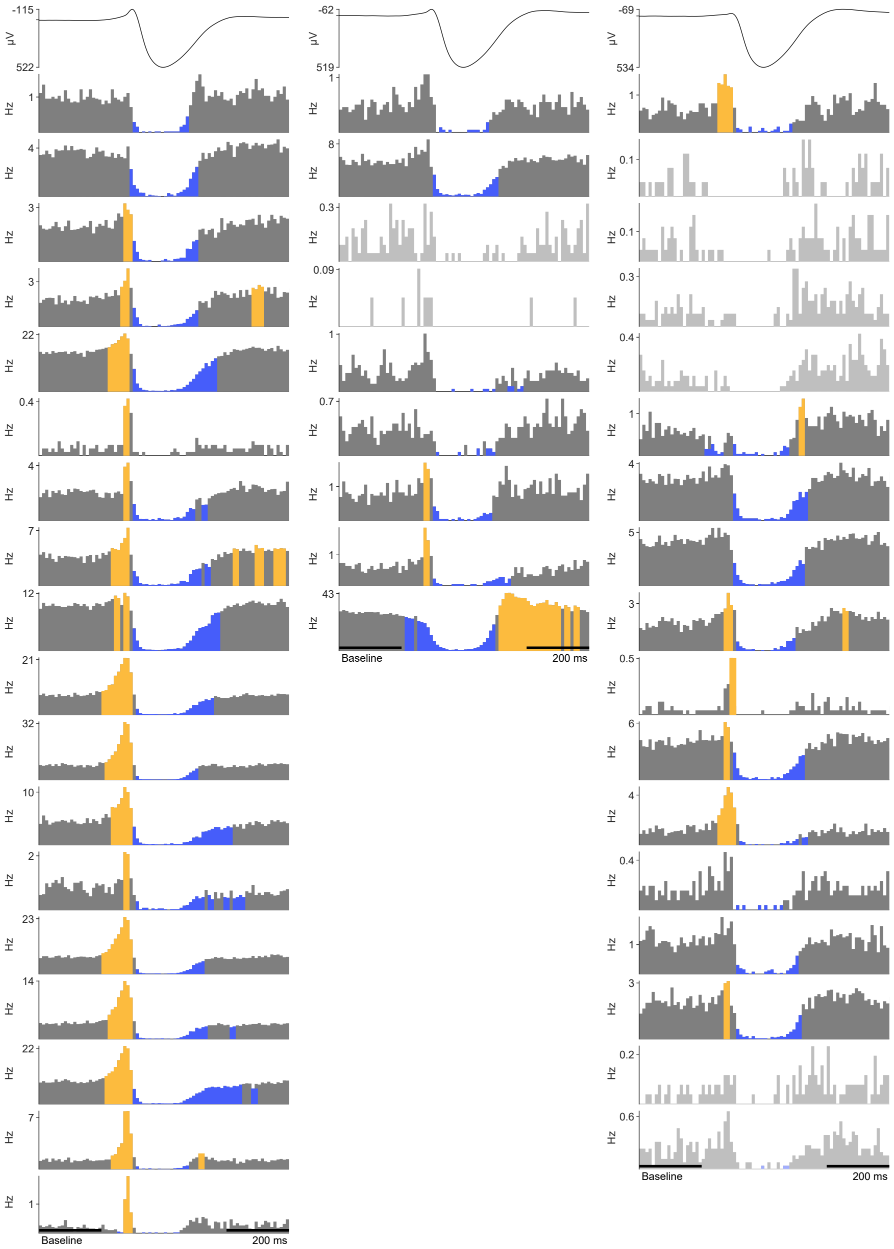

Supplementary Figure 9. Patient 1 (2/3): peri-stimulus time histogram (PSTH) of each unit. Top row shows time-locked local field potential (LFP). Units are organized over nights (columns). A threshold of $p < 0.01$ (first-level t-test) was used to determine contiguous temporal clusters, after which a threshold of $p < 0.05$ (one-sided correction) determined whether the clusters could be explained by permutation (sum of t values, $n = 10.000$) (1). The yellow color indicates significant increases and the blue color decreases compared with the average firing rate during baseline, indicated in the x-axis of bottom left plot.

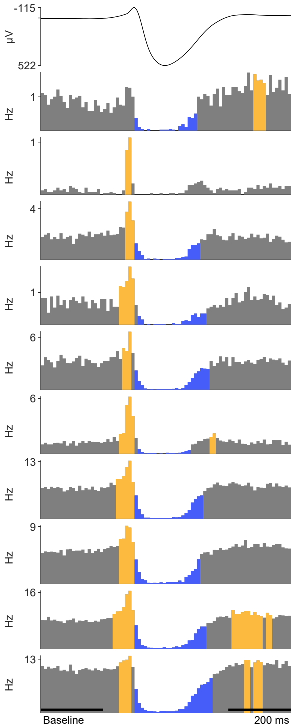

Supplementary Figure 10. Patient 1 (3/3): peri-stimulus time histogram (PSTH) of each unit. Top row shows time-locked local field potential (LFP). Units are organized over nights (columns). A threshold of $p < 0.01$ (first-level t-test) was used to determine contiguous temporal clusters, after which a threshold of $p < 0.05$ (one-sided correction) determined whether the clusters could be explained by permutation (sum of t values, $n = 10.000$) (1). The yellow color indicates significant increases and the blue color decreases compared with the average firing rate during baseline, indicated in the x-axis of bottom left plot.

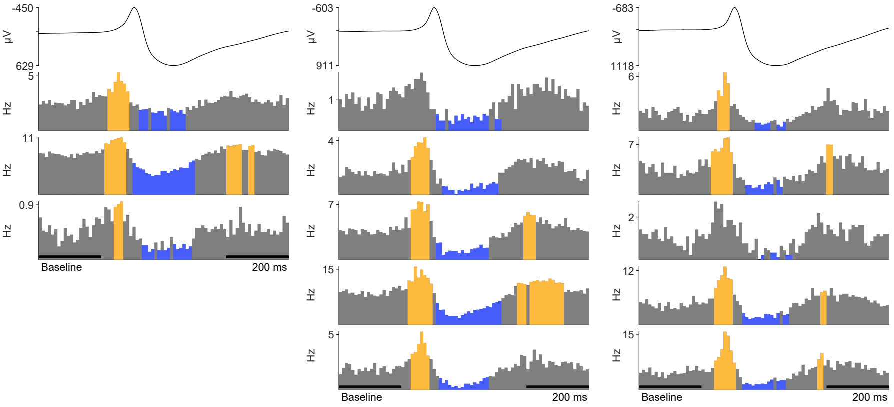

Supplementary Figure 11. Patient 2: peri-stimulus time histogram (PSTH) of each unit. Top row shows time-locked local field potential (LFP). Units are organized over nights (columns). A threshold of $p < 0.01$ (first-level t-test) was used to determine contiguous temporal clusters, after which a threshold of $p < 0.05$ (one-sided correction) determined whether the clusters could be explained by permutation (sum of t values, $n = 10.000$) (1). The yellow color indicates significant increases and the blue color decreases compared with the average firing rate during baseline, indicated in the x-axis of bottom left plot.

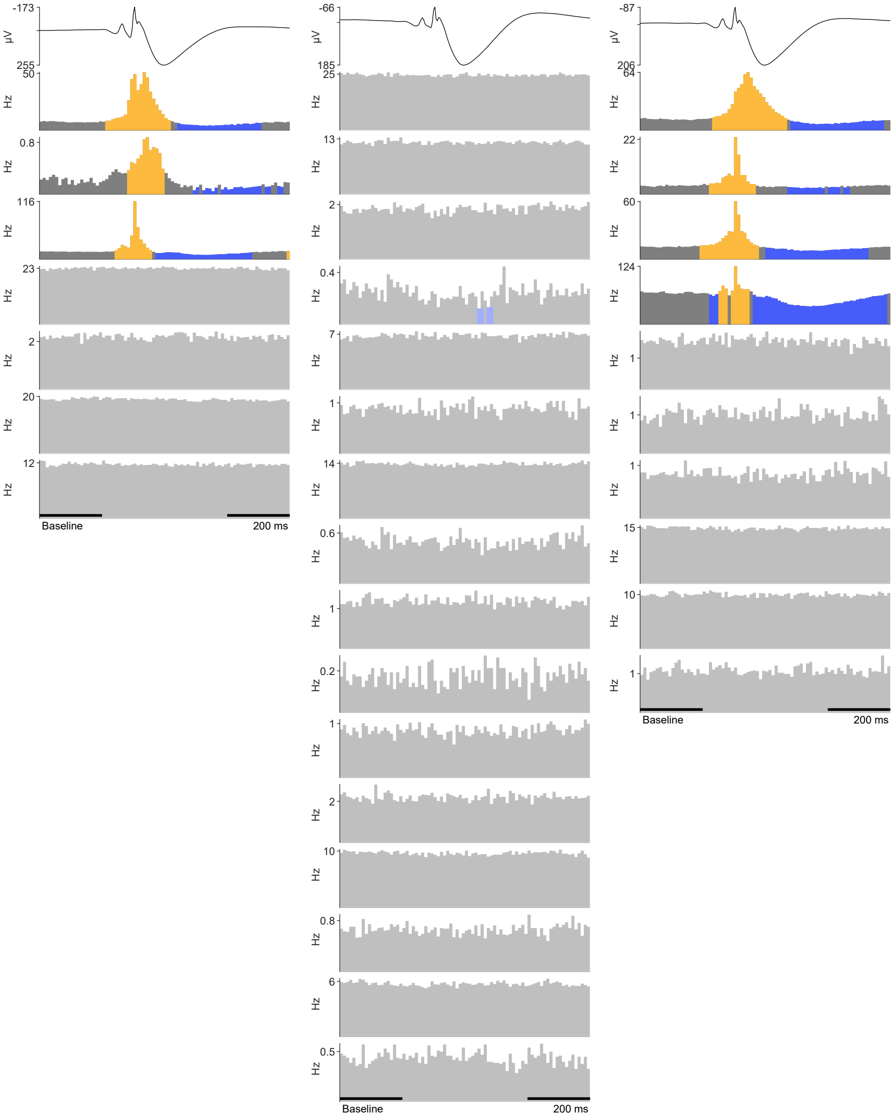

Supplementary Figure 12. Patient 3: peri-stimulus time histogram (PSTH) of each unit. Top row shows time-locked local field potential (LFP). Units are organized over nights (columns). A threshold of $p < 0.01$ (first-level t-test) was used to determine contiguous temporal clusters, after which a threshold of $p < 0.05$ (one-sided correction) determined whether the clusters could be explained by permutation (sum of t values, $n = 10.000$) (1). The yellow color indicates significant increases and the blue color decreases compared with the average firing rate during baseline, indicated in the x-axis of bottom left plot.

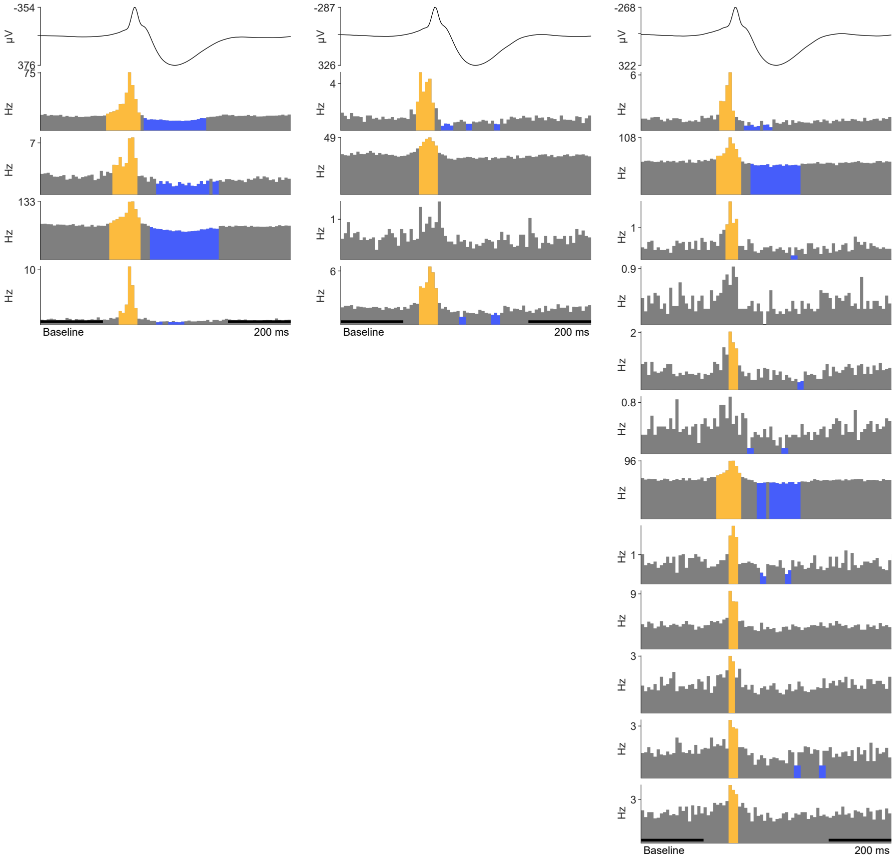

Supplementary Figure 13. Patient 4: peri-stimulus time histogram (PSTH) of each unit. Top row shows time-locked local field potential (LFP). Units are organized over nights (columns). A threshold of $p < 0.01$ (first-level t-test) was used to determine contiguous temporal clusters, after which a threshold of $p < 0.05$ (one-sided correction) determined whether the clusters could be explained by permutation (sum of t values, $n = 10.000$) (1). The yellow color indicates significant increases and the blue color decreases compared with the average firing rate during baseline, indicated in the x-axis of bottom left plot.

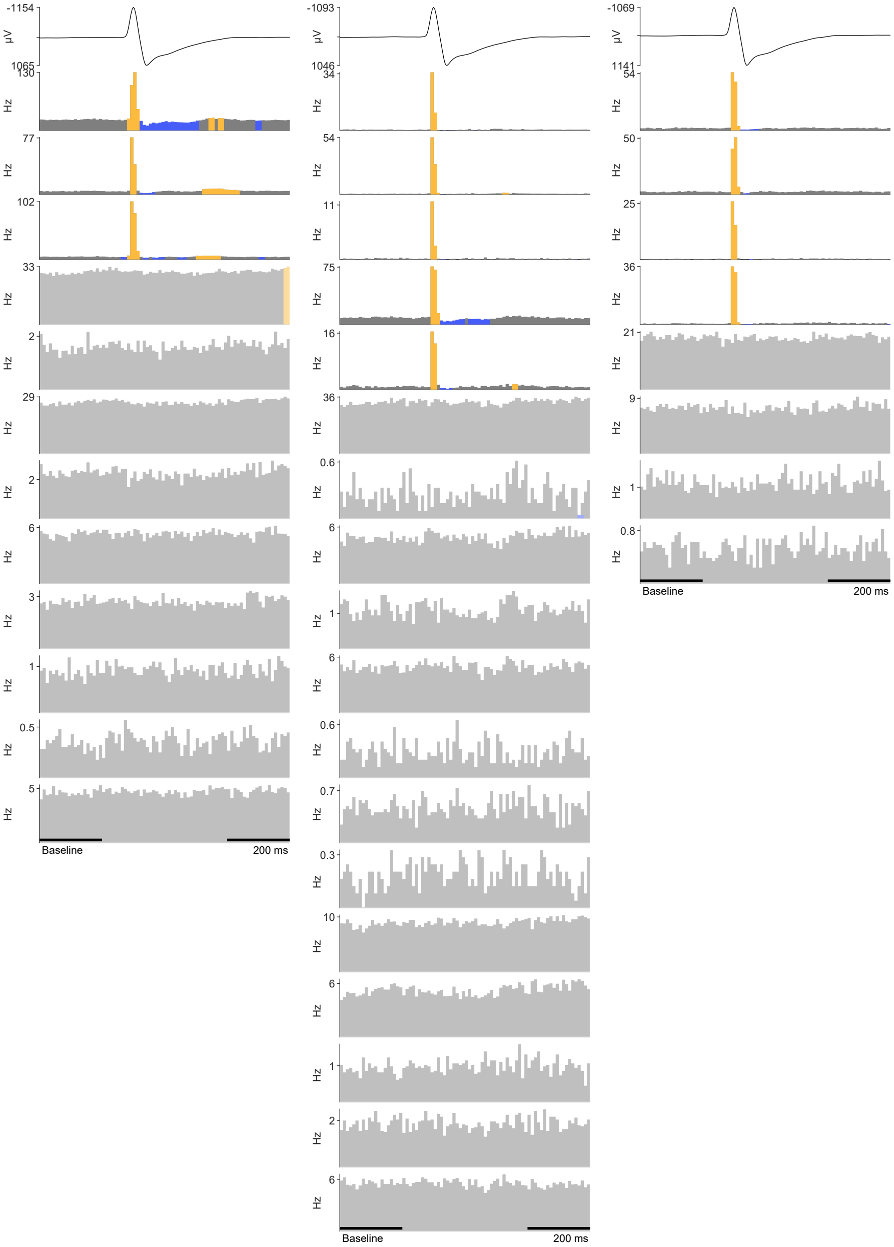

Supplementary Figure 14. Patient 5: peri-stimulus time histogram (PSTH) of each unit. Top row shows time-locked local field potential (LFP). Units are organized over nights (columns). A threshold of $p < 0.01$ (first-level t-test) was used to determine contiguous temporal clusters, after which a threshold of $p < 0.05$ (one-sided correction) determined whether the clusters could be explained by permutation (sum of t values, $n = 10.000$) (1). The yellow color indicates significant increases and the blue color decreases compared with the average firing rate during baseline, indicated in the x-axis of bottom left plot.

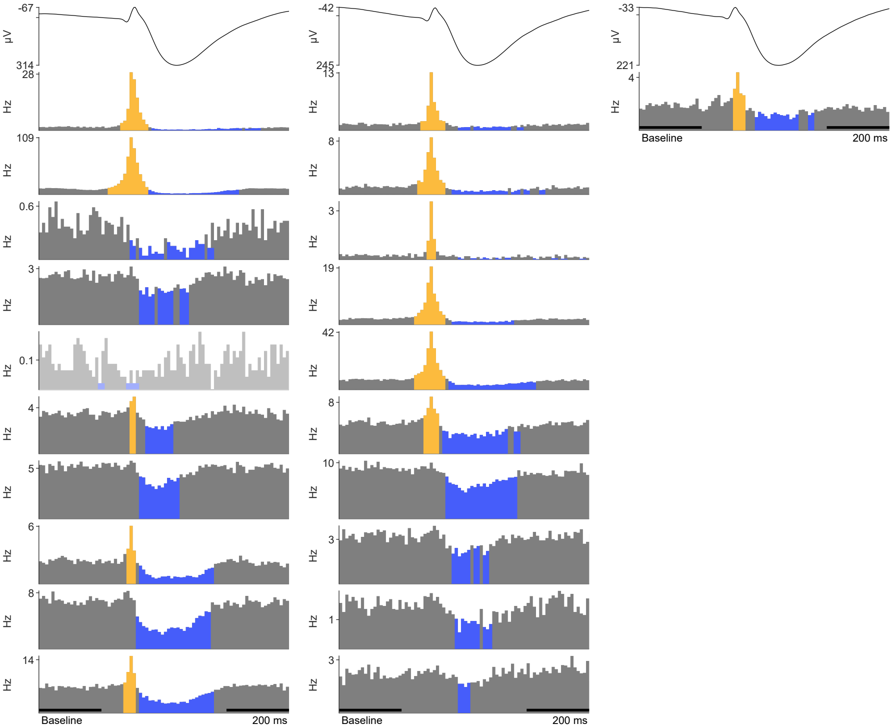

Supplementary Figure 15. Patient 6: peri-stimulus time histogram (PSTH) of each unit. Top row shows time-locked local field potential (LFP). Units are organized over nights (columns). A threshold of $p < 0.01$ (first-level t-test) was used to determine contiguous temporal clusters, after which a threshold of $p < 0.05$ (one-sided correction) determined whether the clusters could be explained by permutation (sum of t values, $n = 10.000$) (1). The yellow color indicates significant increases and the blue color decreases compared with the average firing rate during baseline, indicated in the x-axis of bottom left plot.

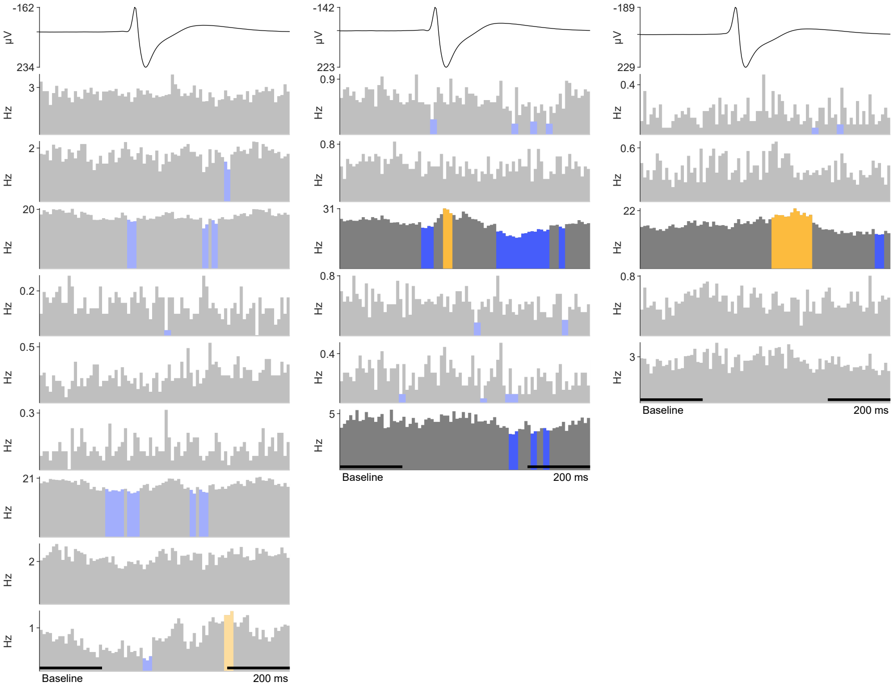

Supplementary Figure 16. Patient 7: peri-stimulus time histogram (PSTH) of each unit. Top row shows time-locked local field potential (LFP). Units are organized over nights (columns). A threshold of $p < 0.01$ (first-level t-test) was used to determine contiguous temporal clusters, after which a threshold of $p < 0.05$ (one-sided correction) determined whether the clusters could be explained by permutation (sum of t values, $n = 10.000$) (1). The yellow color indicates significant increases and the blue color decreases compared with the average firing rate during baseline, indicated in the x-axis of bottom left plot.

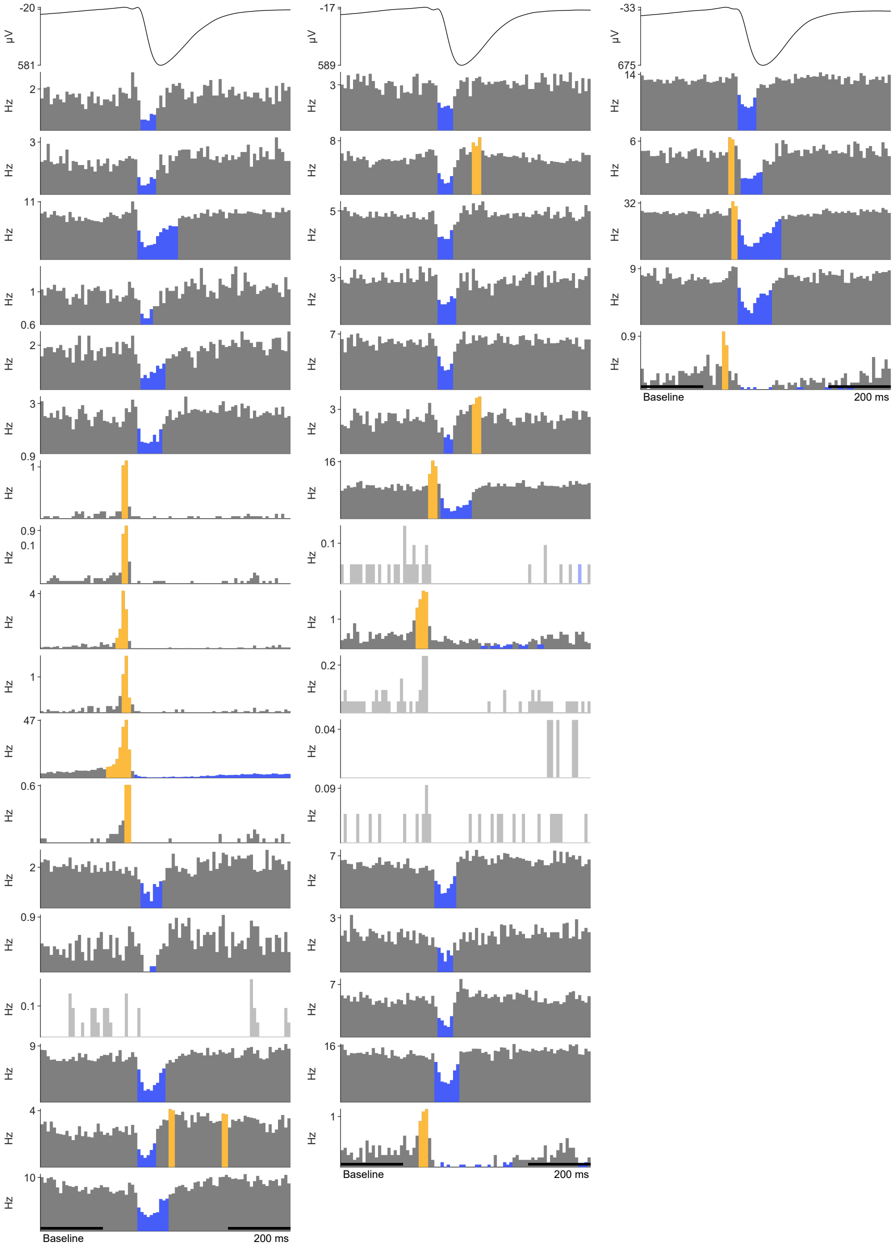

Supplementary Figure 17. Patient 8: peri-stimulus time histogram (PSTH) of each unit. Top row shows time-locked local field potential (LFP). Units are organized over nights (columns). A threshold of $p < 0.01$ (first-level t-test) was used to determine contiguous temporal clusters, after which a threshold of $p < 0.05$ (one-sided correction) determined whether the clusters could be explained by permutation (sum of t values, $n = 10.000$) (1). The yellow color indicates significant increases and the blue color decreases compared with the average firing rate during baseline, indicated in the x-axis of bottom left plot.

References

1. Maris Eric, Oostenveld Robert. Nonparametric Statistical Testing of EEG- and MEG-data Journal of Neuroscience Methods. 2007;164:177–190
